# Comparison of Marine Spatial Planning Methods in Madagascar Demonstrates Value of Alternative Approaches

**DOI:** 10.1371/journal.pone.0028969

**Published:** 2012-02-16

**Authors:** Thomas F. Allnutt, Timothy R. McClanahan, Serge Andréfouët, Merrill Baker, Erwann Lagabrielle, Caleb McClennen, Andry J. M. Rakotomanjaka, Tantely F. Tianarisoa, Reg Watson, Claire Kremen

**Affiliations:** 1 Department of Environmental Science, Policy and Management, University of California, Berkeley, Berkeley, California, United States of America; 2 Marine Programs, Wildlife Conservation Society, Bronx, New York, United States of America; 3 Institut de Recherche pour le Développement, Nouméa Cedex, New Caledonia; 4 Institut de Recherche pour le Développement, Sainte-Clotilde, France; 5 Botany Department, Nelson Mandela Metropolitan University, George, South Africa; 6 Madagascar Program, Wildlife Conservation Society, Antananarivo, Madagascar; 7 Fisheries Center, University of British Columbia, Vancouver, Canada; Swansea University, United Kingdom

## Abstract

The Government of Madagascar plans to increase marine protected area coverage by over one million hectares. To assist this process, we compare four methods for marine spatial planning of Madagascar's west coast. Input data for each method was drawn from the same variables: fishing pressure, exposure to climate change, and biodiversity (habitats, species distributions, biological richness, and biodiversity value). The first method compares visual color classifications of primary variables, the second uses binary combinations of these variables to produce a categorical classification of management actions, the third is a target-based optimization using Marxan, and the fourth is conservation ranking with Zonation. We present results from each method, and compare the latter three approaches for spatial coverage, biodiversity representation, fishing cost and persistence probability. All results included large areas in the north, central, and southern parts of western Madagascar. Achieving 30% representation targets with Marxan required twice the fish catch loss than the categorical method. The categorical classification and Zonation do not consider targets for conservation features. However, when we reduced Marxan targets to 16.3%, matching the representation level of the “strict protection” class of the categorical result, the methods show similar catch losses. The management category portfolio has complete coverage, and presents several management recommendations including strict protection. Zonation produces rapid conservation rankings across large, diverse datasets. Marxan is useful for identifying strict protected areas that meet representation targets, and minimize exposure probabilities for conservation features at low economic cost. We show that methods based on Zonation and a simple combination of variables can produce results comparable to Marxan for species representation and catch losses, demonstrating the value of comparing alternative approaches during initial stages of the planning process. Choosing an appropriate approach ultimately depends on scientific and political factors including representation targets, likelihood of adoption, and persistence goals.

## Introduction

While climate change, overfishing and land use change negatively impact the biodiversity and ecological function of marine ecosystems worldwide [1], [2], increasing evidence shows that effective conservation and management can recover the resource base, conserve biodiversity, and increase fisher's incomes [3], [4], [5], [6], [7]. Yet targeting interventions to “maximize returns” across large regions remains a major challenge: in most tropical seas, for example, basic patterns and interactions among key biological, environmental and social variables remain poorly understood [8]. In addition, while appropriate targeting methods and data sources may exist, they can be underutilized due to a lack of real-world examples and comparative evaluations [9]. In this paper we compare four alternative methods of successive technical complexity for identifying conservation and management priorities across Madagascar's west coast, a regionally and globally important tropical coral reef ecosystem [10], [11].

The waters of the West Coast of Madagascar are home to 90% of Madagascar's coral reefs, large-scale export fisheries for shrimp, octopus, sea cucumbers, and tuna, and important artisanal fisheries. Nonetheless, the formal management of marine resources in the region is in its initial stages. There is only one fully decreed marine protected area (MPA) (Sahamalaza-Isles Radama), with several others in various stages of designation. Less than 1% of Madagascar's reefs are included in no-take areas, the lowest rate for five West Indian Ocean countries [12]. Few additional areas are under formal marine management across a region spanning thousands of kilometers of coastline and home to nearly two million people, many of whom are dependent on marine resources as an important protein source for local consumption and as a source of cash from export or sale [13], [14]. Due to inadequate marine resource management across the majority of the region, fishers target lower trophic levels [15], and the use of illegal and destructive gear such as small-meshed beach seine nets is widespread [16]. As a result, large areas of the region's coral reef ecosystems are chronically stressed [17].

Several governmental, non-governmental and community organizations in Madagascar are interested in increasing the scale of marine resource management on Madagascar's west coast through MPAs and other strategies [18]. In general, increasing the scope and effectiveness of marine resource management across large areas requires a large-scale spatial synthesis of biological, socio-economic and environmental patterns [19]; such a synthesis is lacking across Madagascar's west coast, since the region is large, relatively inaccessible and insufficiently surveyed [11].

Many methods now exist for mapping conservation priorities and management actions in marine systems. Collectively termed “Marine Spatial Planning” [20], this approach may involve traditional techniques such as biogeographic classification [21], gap analysis [22], and “systematic” target-based planning methods [23], [24], [25]. When formulating marine spatial plans, several authors emphasize the need to include socioeconomic as well as biological data [26], [27] and to consider exposure to climate change in the context of persistence of managed areas [28], [29].

Existing examples and reviews of available planning approaches are biased towards marine systems occurring in more economically developed, data rich countries [9], [21], [30], and often focus exclusively on quantitative efficiency measures to identify an “optimal” result [31]. In the planning context within Madagascar, however, uncertainties surrounding potential social and ecological responses to management and threats could lead to lost opportunities, such as sustainable management of fisheries, if there is heavy reliance on a single optimization approach in early planning stages. Therefore, we apply multiple conceptual and analytic techniques to the same data sources to compare results, and provide alternative starting points for developing regional marine conservation and management plans.

## Methods

### Study area

**Figure 1:**
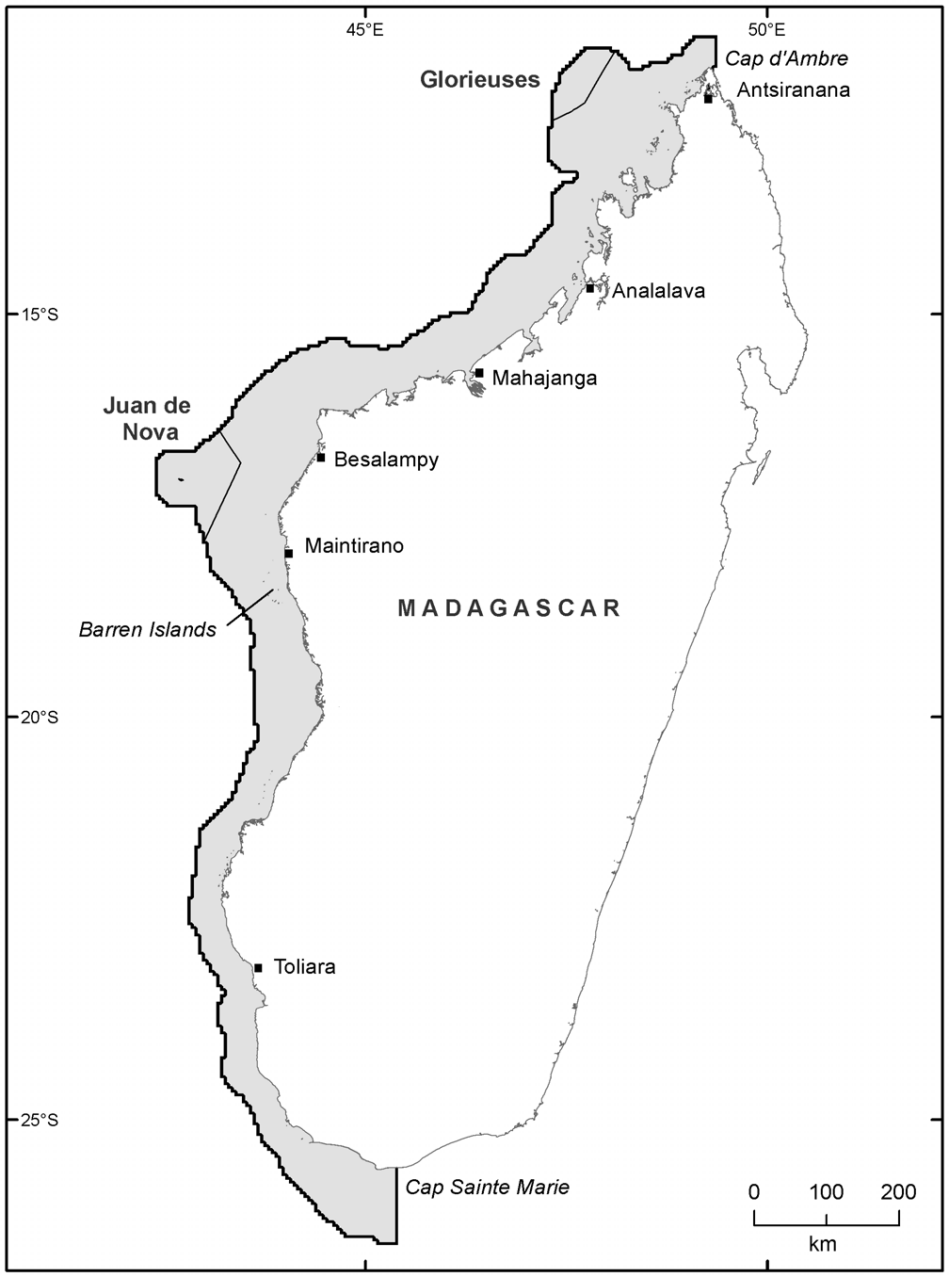
Map of study area on Madagascar's West Coast, and locations mentioned in the text. Study area is shown in grey with black outline Most of the study area is in Madagascar's Exclusive Economic Zone with the exception of small areas that fall in Glorieuses and Juan de Nova.

Our study area is the coastal region of western Madagascar, from Cap Vohimena (Cap Sainte Marie) in the south, to Cap d'Ambre in the north. The region includes extensive fringing, patch and barrier reefs, many small islands, large areas of coastal shelf, mangroves, seagrass beds, and other typical tropical marine ecosystems. Measured on a 1∶250,000 scale [32], the region has 7,000 km of coastline across a 14° latitudinal north-south gradient, and exhibits considerable variation in environmental conditions [33], [34]. To define the study region, we first combined the boundaries of the three coastal bioregions that occur along Madagascar's west coast [18]. These bioregions cover the entire neritic zone from the coastline to a depth of roughly 200 m. As a final step defining the study area, we extended the outer boundary by 0.25 degrees to ensure complete coverage of coastal and neritic habitats, producing a total area of 201,057 km^2^ (Figure 1).

Unlike Madagascar's east coast, most of the west coast has a wide, shallow, gently sloping continental shelf. At the widest point, between Besalampy and Juan de Nova Island, the region is approximately 175 km wide. The narrowest zones are offshore from Toliara province, where faulting has produced a steeper, narrower shelf typically only 40–50 km wide. Ninety-two percent of the study area is within Madagascar's Exclusive Economic Zone (EEZ), adjacent to Mahajanga and parts of Toliara, and Antsiranana provinces. Small portions also fall within the Juan de Nova and Glorieuses EEZ, both territories of France (Figure 1).

According to a recent study of reef geomorphology [35], 90% of Madagascar's coral reefs are found within this study region. These reefs support the highest coral [36] and invertebrate macrofauna [37] species richness in the central and western Indian Ocean, and also have high levels of mollusk and fish diversity [38]. The study area is also important for marine megafauna, including significant populations of five species of marine turtles [39], 27 species of marine mammals, including several threatened species such as dugong (*Dugong dugon*) [40], [41], coelacanth (*Latimeria chalumnae*) [42], as well as a number of regionally important seabird colonies [43], especially for Sooty (*Onychoprion fuscata*) and Crested Terns (*Thalasseus bergii*) [44]. Overall, however, the Indian Ocean is the least known of the tropical seas [45], and the biodiversity of the study area remains poorly surveyed [11]. The large majority of readily available biological data comes from the extensive and now largely degraded reef systems off of Toliara, in the south [46]. The next best-studied area is centered on the island of Nosy Be, 1,200 km to the north. Relatively little is known about the vast area between these two widely separated locations, although recent surveys have begun to specifically target these gaps [47].

Human and environmental impacts on the region are also not well studied but are likely to be highly variable, dependent on the intensity of fishing for subsistence or export, sedimentation from deforestation, and the strength of relatively recent severe sea temperature anomalies [33], [34], [48]. Threats to marine resources are changing with the rapid increase of coastal populations. Two of the west coast's three provinces, Tulear and Mahajanga, now have the highest birth rates in all of Madagscar: 6.3 and 6.1 children per women, respectively [14]. In some areas along the west coast, numbers of boats and fishermen have increased five-fold since the 1980's, contributing to resource decline, particularly in waters near urban centers such as Toliara [17], [46]. Export-driven demand for products such as octopus, sea cucumbers and shark fins is another important driver of ecological impacts. Many of these fisheries show signs of overexploitation, decline, or transition from high- to low-value species [49], [50], [51].

Along the West Coast, Sahamalaza-Isles Radama is the only park established primarily for marine protection that is also fully decreed as an “existing” protected area as stipulated under the Madagascar Protected Areas Law [52], [53]. In addition to this, there are four fully decreed terrestrial protected areas that include some marine or coastal habitats (Kirindy Mitea, Baie de Baly, Lokobe and Nosy Ve). Madagascar protected areas law also includes “temporary” and “new” designations. “Temporary” protected areas have completed an initial administrative process and await final legislative decree to become full protected areas. For “new” protected areas, the administrative procedure for implementation is ongoing [53]. Nosy Hara is the most significant marine area under “temporary” protected status. Since 2009, ten other areas have been designated as “new” protected areas. Along the west coast in total, there are 5,097 km^2^ (9.2% marine, 90.8% terrestrial) of “existing” protected areas, 14,615 km^2^ (10.6% marine, 89.4 km^2^ terrestrial) of “temporary” protected areas, and 4,579 km^2^ (74.8% marine, 25.2% terrestrial) of “new” protected areas. For our study area, this amounts to 0.3% designated as “existing”, 0.9% as “temporary”, and 2.0% as “new” protected areas. The terrestrial portions of reserves are excluded from these percent coverage statistics.

### Spatial data

We collected spatial data for three variables relevant to conservation decisions across the study area: fishing pressure, exposure to thermal stress, and biodiversity (Table 1).

**Table 1:** Data used in the study (I), sources, methods (M), and an overview of the comparisons (C).

**Input variables (I)**	Fishing pressure (catch) (F) [54]Exposure to thermal stress (E) [33]Biodiversity features (B)B1. Bioregions [18]B2. Coral reef geomorphology [35]B3. Mangroves [58], [59]B4. Fish species distributionsB5. Biodiversity value calculated with Zonation algorithmB6. Biological value calculated as species and habitat richness
**Analysis methods (M)**	M1a. Visual gradient overlay of inputs E, F and B5M1b. Visual gradient overlay of inputs E, F, and B6M2. Categorical classification of conservation and management actions from inputs E, F, and B5M3a. Marxan with 30% feature targets from inputs E, F, and B1–B4M3b. Marxan with 16% feature targets from inputs E, F, and B1–B4M4. Zonation with inputs B1–B4 as positively weighted features, and E, F as negatively weighted opposing features
**Comparisons (C)**	C1. Visual comparisons between M1a, M1b, M2 and M3aC2. Quantitative comparisons between M2 (strictest protection category only), M3a, M3b, M4 (threshold at rank with 16.3% average representation):- Total overlap- Average proportion of distributions included (B1–B4)- Fishing pressure in terms of total catch (F)- Exposure to thermal stress (E): Average value, Probability of result missing feature targets

#### Fishing pressure

**Figure 2:**
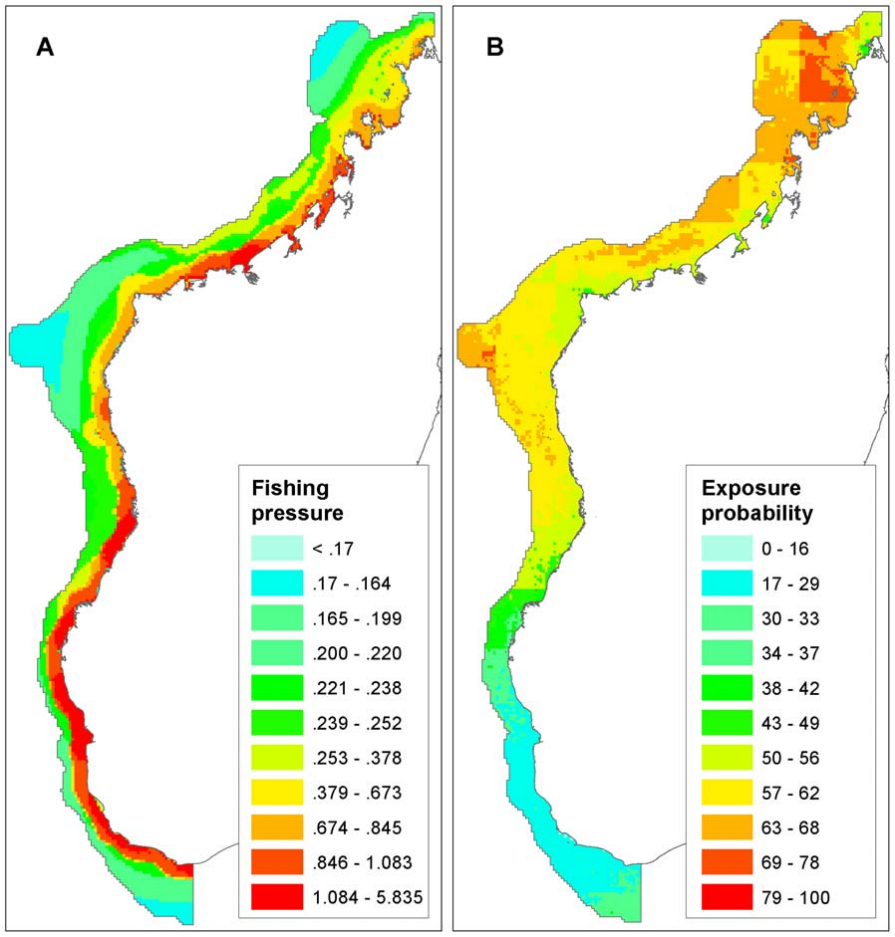
Non-biological inputs to the analysis. A: Fishing pressure from fish catch model [54], units are metric tons/km^2^/yr.; B: Environmental exposure probability [33].

Although there are many human activities in the region, such as tourism and marine transport, the human use with the most significant and direct impact on marine resources in the West Indian Ocean is fishing [45]. Here, we mapped fishing pressure as a combination of motorized and non-motorized coastal fishing pressure (Figure 2a) using a recent model of anthropogenic drivers of marine change for the West Indian Ocean [54]. This model combines spatial data on global fisheries catches [55], [56], tuna purse seine catch data supplied by the Indian Ocean Tuna Commission (IOTC) and data on coastal fisheries derived from national fisheries statistics and population density data.

#### Exposure to thermal stress

Of particular interest is the relative susceptibility of shallow tropical marine regions to heat stress that can cause coral bleaching, particularly under future climate scenarios. For this measure, we used results from Maina et al. [33] to show relative probability of exposure to thermal stress across the study area (Figure 2b). Here, “environmental exposure”, a predictor of the degree to which coral communities are susceptible to climate induced thermal stress, consists of a weighted combination of nine satellite-derived environmental variables [33].

#### Biodiversity

**Figure 3:**
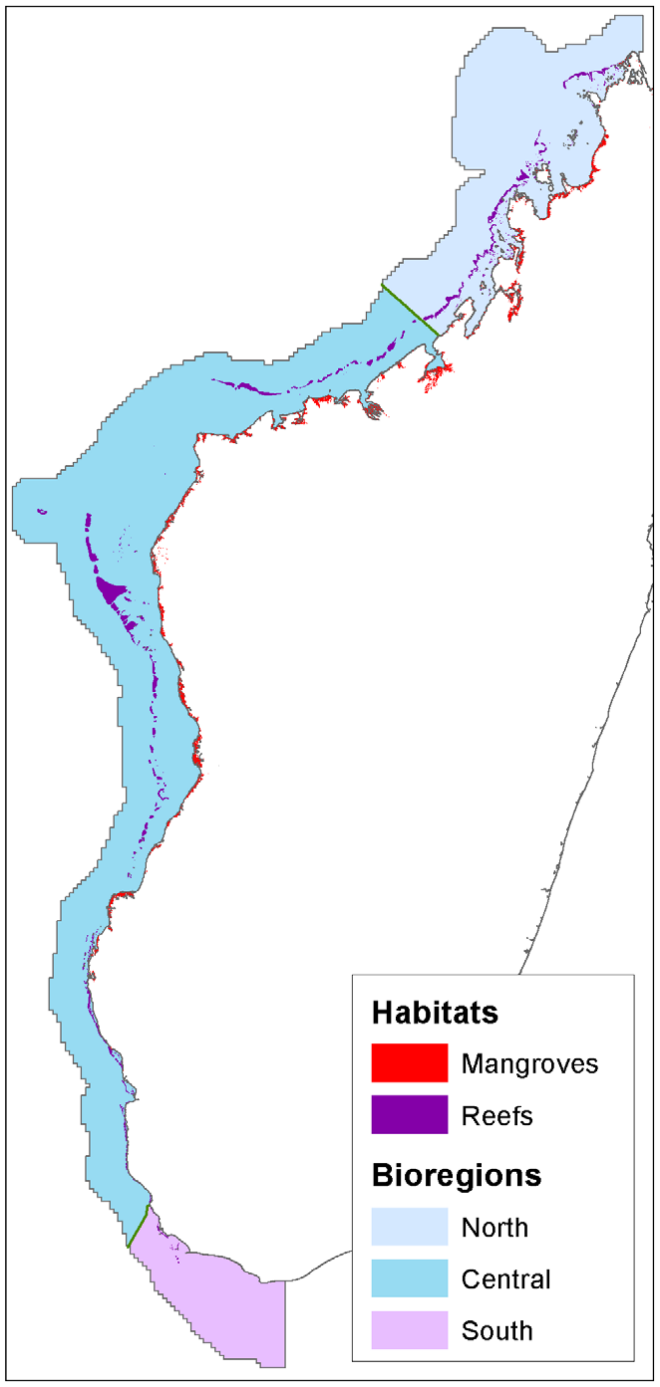
Mangroves, reef geomorphology and bioregions.

**Figure 4:**
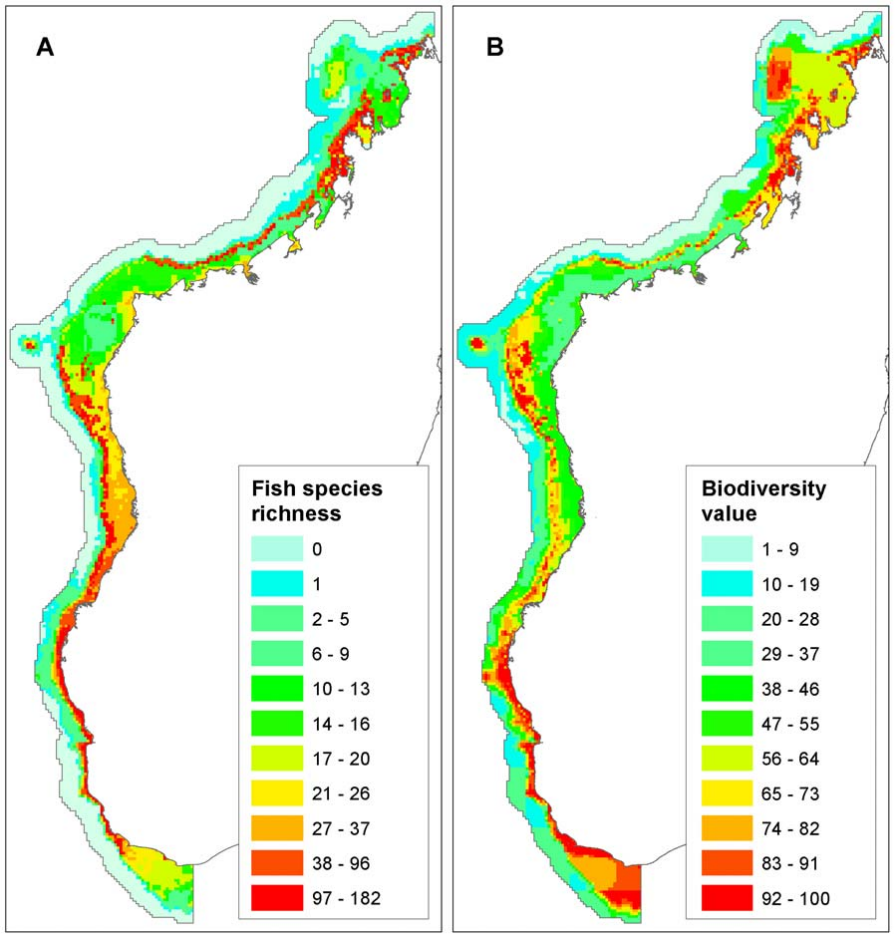
Additional biological inputs into the analysis. A: Biological richness showing number of fish species per 25 km^2^ grid cell; B: Biodiversity value measured using Zonation algorithm.

We collected spatial distribution data on four biodiversity features: bioregions, coral reefs, mangroves and fish species distributions (Figure 3, Figures 4a,b, Table 2). Bioregions are broad units of relatively similar biological and environmental conditions, frequently used to ensure representation within reserve networks [57]. We utilized a map of bioregions developed from species data, environmental data and expert opinion from a recent West Indian Ocean conservation planning exercise [18]. This map recognizes three coastal bioregions across the entire neritic zone of western Madagascar's coast: a north-western unit from Cap d'Ambre to 15 km north of the city of Mahajanga; a large central unit extending from north of Mahajanga to 150 km south of Toliara; and finally a southern unit continuing from south of Toliara around Cap Vohimena. We created a map of mangroves from a simple union of two sources: Harper et al. [58] and Moat & Smith [59]. Both authors used Landsat TM imagery from 1998 to 2005 to map mangroves at approximately 30 m resolution. We took reef geomorphological types from a West Indian Ocean map of reef morphology produced by Andréfouët et al. [35] from Landsat imagery acquired between 1999 and 2003. The reef data includes 16 reef geomorphological types within the study area.

**Table 2:** Biodiversity data used in the analysis.

Data type	Dataset	Source
**Bioregions**	3 West Coast Madagascar Bioregions	Indian Ocean Commission [18]
**Habitats**	Mangroves	Harper et al. [58]; Moat & Smith [59]
	Reef geomorphology: Atoll rim	Andréfouët et al. [35]
	Reef geomorphology: Bank barrier	Andréfouët et al. [35]
	Reef geomorphology: Bank lagoon	Andréfouët et al. [35]
	Reef geomorphology: Coastal barrier reef complex	Andréfouët et al. [35]
	Reef geomorphology: Coastal/fringing patch	Andréfouët et al. [35]
	Reef geomorphology: Continental lagoon	Andréfouët et al. [35]
	Reef geomorphology: Diffuse fringing	Andréfouët et al. [35]
	Reef geomorphology: Fringing of coastal barrier complex	Andréfouët et al. [35]
	Reef geomorphology: Intra-lagoon patch-reef complex	Andréfouët et al. [35]
	Reef geomorphology: Intra-seas exposed fringing	Andréfouët et al. [35]
	Reef geomorphology: Intra-seas patch-reef complex	Andréfouët et al. [35]
	Reef geomorphology: Lagoon exposed fringing	Andréfouët et al. [35]
	Reef geomorphology: Ocean exposed fringing	Andréfouët et al. [35]
	Reef geomorphology: Outer barrier reef complex	Andréfouët et al. [35]
	Reef geomorphology: Shelf patch-reef complex	Andréfouët et al. [35]
	Reef geomorphology: Shelf slope	Andréfouët et al. [35]
**Species**	251 fish species models generated using MaxEnt	REBIOMA, unpubl. data

To map fish distributions, we started with a list of 530 focal fish species identified by Malagasy and international marine conservation experts as conservation priorities in Madagascar. From this list we collected available locality data from private and public sources (Table 2), including the Global Biodiversity Information Facility (http://data.gbif.org) and the Ocean Biogeographic Information System (http://www.iobis.org). We then used MaxEnt software [60] to model fish species distributions across the planning region for the 274 species (Table S1) with at least eight non-duplicate records (Methods in Materials S1), a common minimum threshold [61]. Environmental data in MaxEnt models consisted of the following nine environmental datasets with an original resolution between 1 km^2^ and 1 degree: chlorophyll [33], current velocity [33], photosynthetically active radiation [33], sea-surface temperature [33], ultraviolet irradiance [33], wind speed [33], salinity [62], depth [63], and percent reef [35].

In choosing species and habitats for the analysis, we selected those that were most important biologically (e.g. rare, threatened), important as providers of ecosystem services (e.g. keystone habitats), and important culturally (e.g. for fisheries). Unfortunately, this selection was necessarily limited by data availability. We could not include maps of sea-grass distributions, for example, despite the critical importance of this habitat for regional ecosystem services, such as fisheries and nodes for connectivity [64].

We resampled all grids to 25 km^2^ for use in MaxEnt and subsequent analyses. Although it is possible to resample to a smaller grid, we felt this is the minimum resolution that is likely to reliably reflect patterns of biodiversity, exposure and resource use across the study area given the current understanding and resolution of the data.

We used the biodiversity features maps to measure biodiversity value in two distinct ways. One, we calculated biological richness as the sum total of fish species in each 25 km^2^ cell. To determine fish species presence, we first applied a threshold to the fish species distribution models to convert them to binary presence-absence maps. We used a fixed logistic threshold value of 0.75, which conservatively selects environmental conditions where fish are highly likely to be present. Biological richness is a common measurement of biodiversity value that is widely used for prioritizing conservation areas [65]. Two, we used the benefit-function conservation priority-setting software Zonation [66] to map biodiversity value of each cell (Methods in Materials S1). Zonation uses principles of complementarity to produce continuous ranking of biodiversity value from input species distribution models, habitats and bioregions, so that selection of any subset of top-ranked grid cells (e.g. the top quartile) maximizes species, habitat, and bioregion representation within the subset.

### Mapping conservation and management priorities

We conducted four separate analyses of the same primary datasets (e.g. fishing pressure, exposure to thermal stress and biodiversity) (Table 1): 1) Visual gradient overlay of these three primary datasets layers in red, green, and blue (RGB) color space; 2) categorical classification of proposed conservation and management action zones; 3) target-based site optimization algorithm using Marxan, the most widely used target-based marine conservation planning optimization method [24], [67]; and finally, 4) conservation priority ranking with Zonation, an algorithm that produces a continuous measure of conservation value [68].

#### Visual gradient overlay in RGB color space

To map each of the three variables in continuous RGB space, we first converted the original data values to a 0–255 scale, so as to render them as 8-bit TIFF files in the ENVI image processing software [69]. Next, we displayed the three variables together as single bands in a 3-band RGB composite image, with biodiversity value assigned to the red band, fishing pressure assigned to green, and environmental exposure assigned to blue. We produced two maps, the first with biodiversity value equal to richness, and the second with biodiversity value mapped by Zonation. By default, ENVI applies a 2% linear stretch to images, which facilitates interpretation of color patterns. The value or intensity of each primary color indicates the relative strength of that variable in that location. In addition, the additive combinations of the primary colors show how the three variables interact across the study area. Areas dominated by blue, for example, show where exposure is the dominant variable; areas dominated by red show where biodiversity is stronger than fishing pressure or exposure, and so on.

#### Categorical classification of proposed conservation and management action zones

We built on conservation action frameworks applied recently to coral reefs and fishing landing sites in five countries [12], [70] to classify the study region based on binary splits and combinations of the three variables. First, we converted the continuous 25 km^2^ measures of biodiversity value measured by Zonation, fishing pressure and exposure to binary “low” and “high” values, defining “high” as the top quartile of values, and “low” as the bottom three quartiles for each input variable. Next, we combined the three binary grids to produce a single map with eight classes representing all unique combinations of the “high” and “low” values. Finally, we proposed a conservation and management action category for each class based on these unique attributes [12], [70]. For example, in areas characterized by high biodiversity value, low fishing pressure and low exposure, we recommended a management regime that emphasizes large closures and strict protection, as these waters are likely to contain biodiversity of global significance, along with a high probability of persistence due to low climate impacts and conflicts with fishing pressure.

#### Marxan target-based site optimization algorithm

Marxan maps areas that meet quantitative representation targets for conservation features (in our case the distribution maps for fish, habitats, and bioregions), while minimizing an overall objective function score that can include different types of costs [24]. Here, we configured the objective function to penalize failure to meet feature targets, and minimize boundary length and total costs of the selected areas (Methods in Materials S1). We selected a feature representation target of 30% for all conservation features. 30% is a common representation level for marine conservation planning [71], and matches the representation target recommended at a recent Indian Ocean Commission Protected Area Network (IOCPAN) conservation planning workshop [18]. Since Marxan is generally run with only binary (presence-absence) species distribution models as an input, for fish species, we used the previously described presence-absence species distribution models where thresholds were established. We used the fish catch data to assign an economic value or selection “cost” to each 25 km^2^ planning unit. We also used a feature of Marxan that allows the inclusion of an additional probability-based cost for each planning unit, typically representing the likelihood of a threatening process, such as catastrophic bleaching [28]. We used the environmental exposure data [33] directly as this measure of future threat probability. By minimizing this set of terms, Marxan attempts to identify reserve systems that meet 30% targets for conservation features, minimize total boundary length and costs, and maximize the probability that conservation features will persist in the face of future threats. We ran Marxan 100 times, selecting the “best” run (i.e. the one with the lowest overall objective function score) as our result.

#### Zonation conservation priority ranking

The most recent Zonation release (version 3.0.2) allows input features to be positively or negatively weighted. This enables the inclusion of both positively weighted features representing biodiversity, as well as negatively weighted features to be avoided, such as exposure or other threatening conditions. For this analysis, we input continuous fish species distribution models, habitat maps, and bioregions as positively weighted features, and fishing pressure and exposure to thermal stress as negatively weighted features (Methods in Materials S1). In all other respects, this Zonation analysis (hereafter “weighted Zonation”) was similar to the one described previously.

#### Comparisons

We compared the Marxan result with the strictest protection category of the categorical classification result (hereafter “large closures” result) and the weighted Zonation, as all three primarily represent areas of high biodiversity value, low cost, and low exposure probability. We made four comparisons of these three results. First, we calculated the total overlap between the three results. Second, we compared the average proportion of conservation features included in each result (Methods in Materials S1). Third, we compared total fish catch included in each result. This is the opportunity cost of withdrawing these areas from production, and serves as a measure of marine reserve efficiency [72]. Fourth, we compared the predicted persistence of each result in terms of exposure probability, in two ways: first by looking at exposure values per grid cell across each result, and second using the value reported by Marxan that shows the probability of a solution missing its conservation feature targets [73]. Although this is a Marxan-specific measure, we locked the other results into runs of Marxan to be able to compare this value (Methods in Materials S1). Finally, we ran Marxan a second time. Here, instead of setting feature representation targets to 30% for conservation features, we used a target equal to the average proportion of species and habitat distributions included in the large closures map, and again compared overlap, average proportion of species ranges included, opportunity cost, and exposure. Similarly, because Zonation produces a continuous ranking of biodiversity value, we selected top ranked cells of the weighted Zonation result until the average proportion of species and habitats represented matched that of the large closures map, and set this as a threshold to produce a binary Zonation result. This thresholded Zonation result (hereafter “Zonation 16%”) and the second Marxan analysis facilitate comparison of these results to the large closures map by ensuring that each achieves approximately the same representation targets.

## Results

We produced two maps of biodiversity value, one based on the estimated numbers of fish species (Figure 4a) and one with biodiversity value calculated by the first run of the Zonation algorithm on biodiversity features alone (Figure 4b). The fish species richness map shows that areas of high species richness are concentrated on the fringing reefs of the southwest, the barrier reefs off Maintarano and Besalampy, Juan de Nova, and the fringing reefs, islands and atolls of the Northwest (Figure 4a). The map produced by Zonation highlights many of these same areas for high biodiversity value (Figure 4b), with some key differences, notably higher estimates for the Banc de Leven some 40 km west-northwest of the Nosy Mitsio group, and the large shallow banks southwest of Cap Sainte Marie.

**Figure 5:**
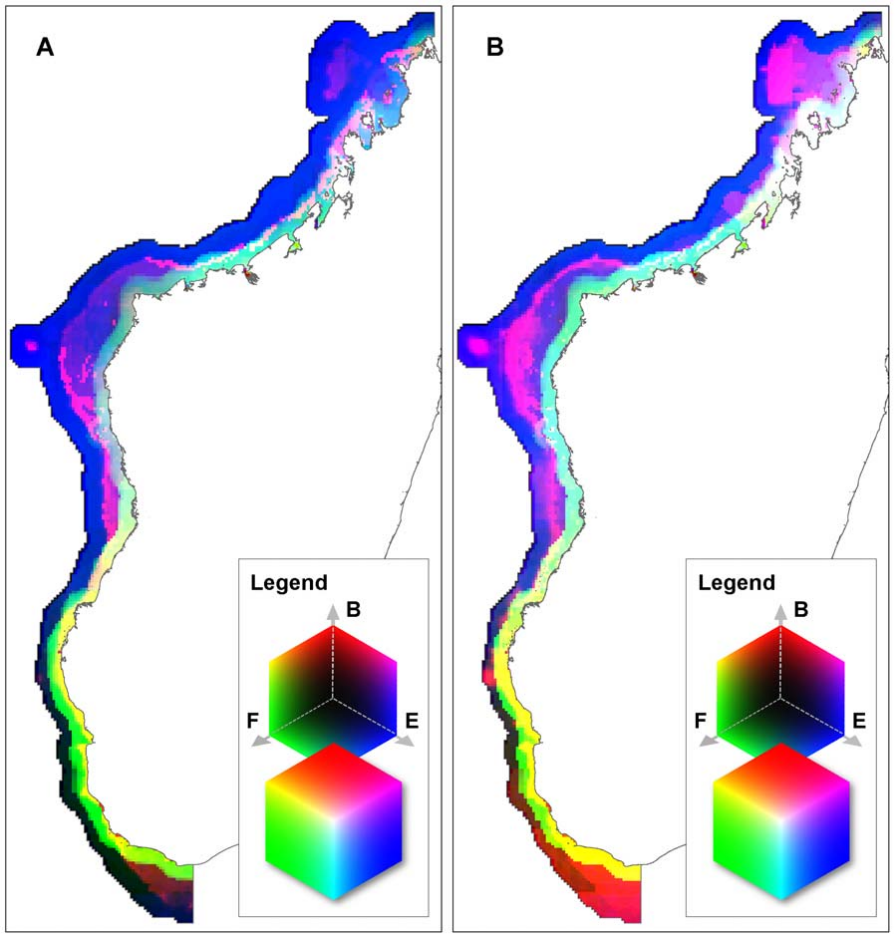
Two results of RGB visual overlay of primary variables (biodiversity, fishing pressure, exposure). A: Biodiversity value expressed as fish species richness; B: Biodiversity value measured using the Zonation algorithm. Key shows classification in 3-dimensional RGB color cube, with biodiversity (letter B in the key) assigned to Red (z-axis), fishing (F) assigned to Green (y-axis), and exposure (E) assigned to Blue (x-axis). Only the colors formed on the inner and outer planes of the cube are visible. On the inner planes, one variable is always 0. On the outer planes, one variable is always 255. The inner corner (black) has 0 values for all variables. The outer corner (white) has values of 255 for all variables.

The visual overlay of the use, exposure, and biodiversity in RGB color space provides a visual interpretation of the relative strength of each of the three primary variables – use represented by green, biodiversity by red, and exposure by blue (Figures 5a,b). For example, in both results, large areas of the near-coastal southwest are yellow, indicating both high biodiversity and fishing, but low exposure, while the central coastal areas are light-blue indicating high exposure and fishing, but low biodiversity value. The far west is dark blue where exposure is the dominant variable; and the large, near-coastal areas of the northwest trend towards white, indicating a saturation of all three variables. These two maps also facilitate the comparison of the two alterative biodiversity measures, by showing how they interact with the non-biological variables.

**Figure 6:**
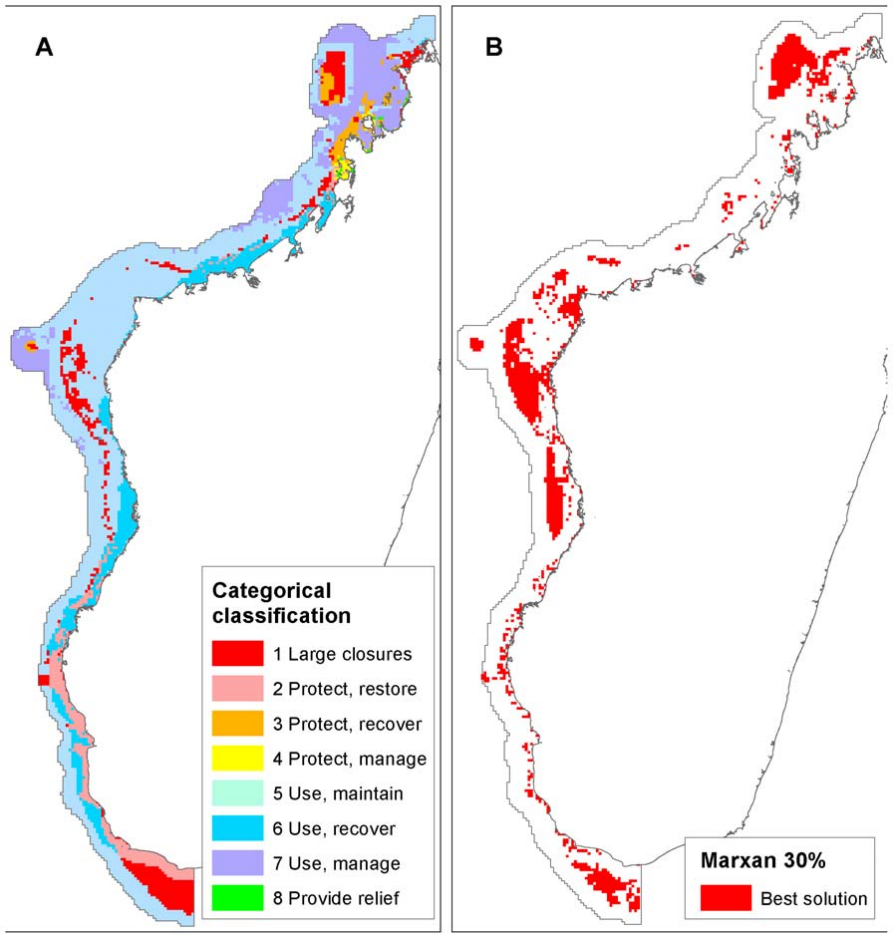
Two views of conservation and management priorities. A: results of the categorical classification (see Table 3 for class descriptions); B: target-based priority-setting with Marxan.

The result of the categorical management action classification extends this simple visual classification to assign a conservation management priority along a gradient of protection, use and exposure risk corresponding to the eight possible combinations of high and low biodiversity, fishing and exposure values (Figure 6a; Table 3). By far the largest area is within the sustainable use and maintain category (Class 5: 87,682 km^2^), which is made up of relatively deep, offshore habitats, where all three variables are low; hence conditions are suitable for managing use up to sustainable limits. This class includes 49.7% of the total study area, and highlights the fact that the majority of the study area (81.0%) maps to the lowest quartile of environmental exposure. In contrast, only 23.7% of the area maps to high biodiversity value (classes 1–4). All of the smaller size classes have high exposure, with a mix of biodiversity and fishing pressure. The smallest class 8 (412 km^2^), suggests providing relief to local communities, is orders of magnitude smaller than the largest, and is characterized by high exposure and fishing pressure, and low biodiversity value.

**Table 3:** Management categories for management actions assigned to combinations of high and low variables based on the conceptual model of McClanahan et al. [68].

Class	Area (km^2^)	Exposure	Fishing pressure	Biodiversity Value	Category	Description
1	20,528	Low	Low	High	Large closures	Maintain high biodiversity values through strategies including large closures, strict protection and minimal use where possible
2	14,622	Low	High	High	Protect and restore	Maintain and restore high biodiversity values through closures, strict protection, reduced use and active restoration
3	5,314	High	Low	High	Protect and recover	Protection, recovery and restoration necessary to maintain high biodiversity values with a degree of resignation to ecological degradation: management involves risk of failure due to high exposure
4	1,357	High	High	High	Protect and manage	Protection, recovery and restoration necessary to maintain high biodiversity values. High risk of failure due to high exposure and use
5	87,682	Low	Low	Low	Use sustainably and maintain	Manage towards limits of sustainable use. Lower biodiversity values, but lower risk due to lower exposure and use; overuse to extinction unlikely
6	19,426	Low	High	Low	Use sustainably and recover	Manage towards limits of sustainable use with recovery and restoration, reduce use towards the limits of sustainable yield
7	27,018	High	Low	Low	Use sustainably and manage	Manage towards limits with some resignation to ecological degradation; management interventions carry risk of failure due to high exposure, and low returns due to low biodiversity value.
8	412	High	High	Low	Provide relief	Communities here may require relief because of high existing fishing pressure and uncertain future resilience and sustainability

Total area sums to less than the study area (86%) because of the extension of the study area by 0.25 degrees inland.

The Marxan 30% target result shows areas that meet quantitative conservation targets at minimum cost and high probability of persistence (Figure 6b). Results are distinctly clustered within the south, central and northern parts of the study area, with a scattering of individual planning units selected in between. By far the least selected areas are in the northwest between Cap d'Ambre and Analalava, where exposure probability and, therefore, the probability of persistence is low, and fishing pressure is also relatively high.

**Figure 7:**
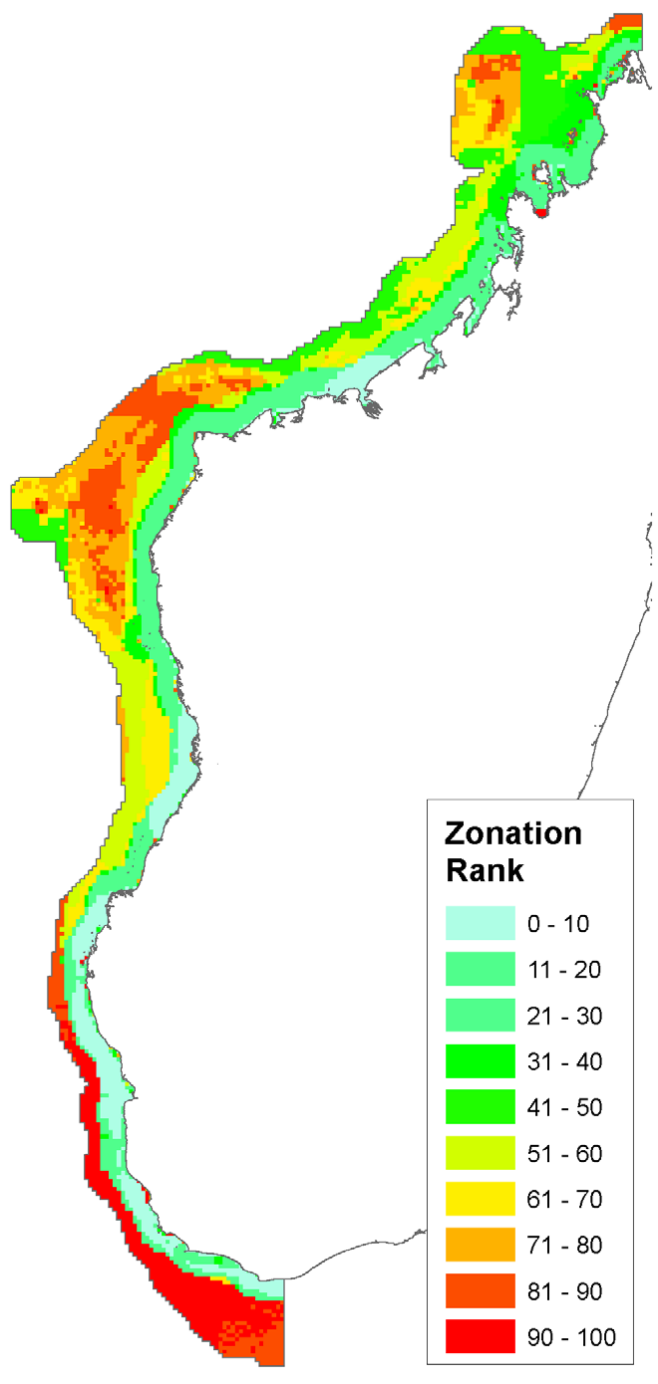
Weighted Zonation result. This map shows a continuous ranking of conservation value by the Zonation algorithm. Higher ranked cells are more important for species representation, and tend to have lower fishing pressure and exposure values.

The weighted Zonation result shows all areas ranked in order of their conservation value (Figure 7). Because this run of Zonation tends to avoid negatively weighted features by giving them a low ranking (here fishing pressure and exposure), the highest ranked areas are generally those furthest away from where the negatively weighted variables have their strongest influence. As a consequence, the dominant concentrations of high ranked cells are found in three places: offshore southwest, offshore central in the vicinity of Juan de Nova, the Barren Islands and associated barrier reefs, and offshore northwest, on the Banc de Leven.

Comparing the management actions categorization and Marxan results, we find that the large closures result covers 20,528 km^2^, and the Marxan 30% result covers 39,585 km^2^. The large closures result shares 61.1% of its selected area with the Marxan result; 32.0% of the Marxan result overlaps with the large closures result. The Marxan result includes an average of 28.9% of the total distribution of each species or habitat. The large closures result includes 16.3% of the total distribution of each species on average, which is significantly less than the Marxan result (two-sample paired t-test, t(590) = 18.4655, p<0 .0001). Finally, assuming fishing is excluded entirely from the selected areas and there are no spillover benefits, the impact of the large closures categorization is 6,340.2 tons of fish/year, compared to 14,410.9 tons/year for Marxan. After reducing feature targets in Marxan to 16.3% (hereafter Marxan 16% result) to match the average proportion of the range captured by the large closures result, we find the Marxan 16% result covers a comparable area (21,266 km^2^), and there is no longer a significant difference between species proportions represented (Marxan 16% result: 15.4% of each species range, two-sample paired t-test, t(590) = −1.2875, p = 0.19) (Table 4). The large closures result shares 38.8% of its selected area with the Marxan 16% result, while 37.4% of Marxan 16% result overlaps with the large closures result. We find the fisheries impact of the Marxan 16% result is 6,393.6 tons/year, 56.0% less than included in the Marxan 30% target result (Table 4), and similar to the large closures result.

**Table 4:** Comparison of results.

Result	Area (km^2^)	Average total distribution represented	Fishing cost (tons/year)	Mean exposure probability	Probability result misses targets
Categorical classification “large closures”	20,528	16.3%	6,340.2	0.47	0.26
Marxan with 30 percent targets	39,585	28.9%	14,410.9	0.52	0.07
Marxan with 16 percent targets	21,266	15.4%	6,393.6	0.52	0.14
Zonation top ranked cells	33,516	16.3%	6,580.8	0.38	0.29

**Figure 8:**
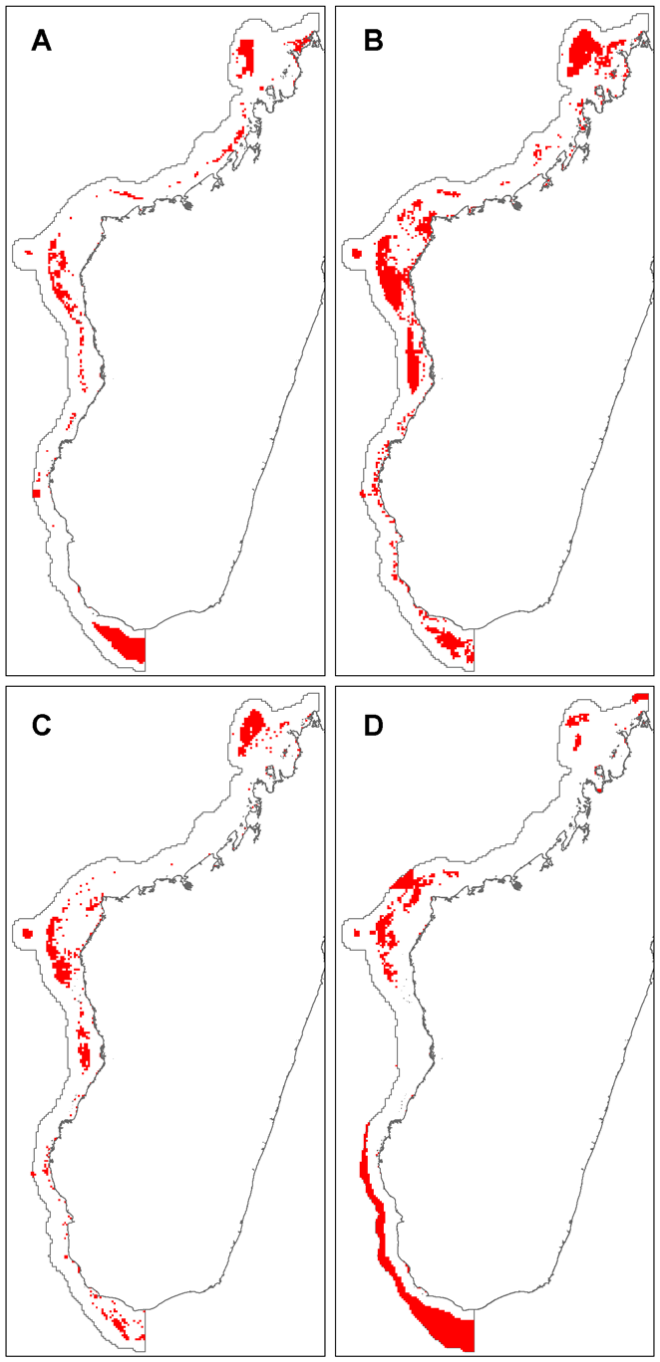
Comparison of results. A: Strict protection class of the categorical classification; B: Marxan 30% solution; C: Marxan 16% solution; D: Zonation 16% solution.

**Figure 9:**
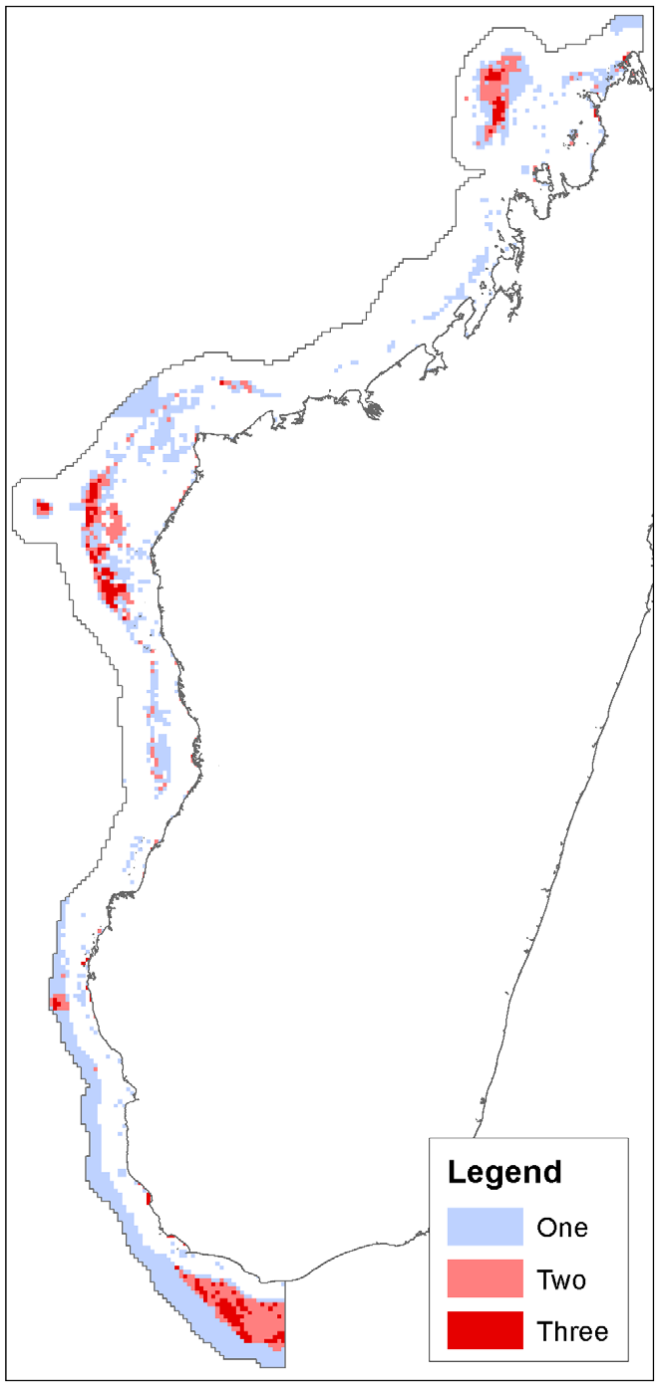
Overlap between results. The number (one, two or three) indicates the number of 16% solutions represented; in other words, the number of times a planning unit has been selected by either the strict protection, Marxan 16% or Zonation 16% result.

Comparing the Zonation 16% result to the other results, we find that this thresholded result covers 33,516 km^2^, and has a predicted fisheries impact of 6,580.8 tons/year, and is therefore larger in area and fisheries impact than the other two results that represent about 16% of species' ranges on average, but smaller in both respects than the Marxan 30% result (Table 4). The mean proportion of ranges represented (16.3%) is not significantly different from the other results (Marxan 16% result: mean 15.4% of each species range, two-sample paired t-test, t(590) = −1.2582, p = 0.2088; Large closures result: mean 16.3% of each species range, t(590) = −6e-04, p = .9995). The Zonation 16% result shares 21.4% and 31.7% of its selected area with Marxan 16% and the large closures result, respectively. The Marxan 16% result and the large closures result, on the other hand, share 33.7% and 51.8% of their selected area with the Zonation 16% result (Table 4, Figures 8–9).

Finally, we find that the distribution of exposure values in the large closures result is significantly lower than in both Marxan results (Wilcoxon rank-sum test, p<0.0001, two-tailed). The Zonation 16% result has the lowest mean exposure values, and this distribution is also significantly different from all other results (Wilcoxon rank-sum test, p<0.0001, two-tailed). The probability of missing conservation feature targets due to exposure, however, is lower in both Marxan results and highest in the Zonation 16% result (Zonation 16%: 0.29, Large closures: 0.26, Marxan 16%: 0.14, Marxan 30%: 0.07, Table 4).

## Discussion

We had two primary aims with this work. First, we applied several different methods for mapping conservation management options across a relatively vast, poorly known region, to see if broad trends emerge irrespective of approach. Second, we evaluated the conditions where alternative approaches might provide results comparable to methods using optimization. Overall, despite the different methods employed, the results show substantial similarities (Figures 5, 6, 7, 8, 9). For example, many areas dominated by red and purple in the continuous RBG color space results (areas of high biodiversity value) were also identified in the Marxan, large closures, and weighted Zonation results. The most prominent examples are the Barren Islands and associated barrier reef systems north and east of Maintirano, and the Juan de Nova Island reefs. Two additional large areas, the Banc de Leven to the northwest, and the large shallow banks southwest of Cap Sainte Marie, are highlighted as conservation priorities in all results with the exception of the RGB map, which used species richness as the measure of biodiversity value (Fig. 4a). Species richness is the only biodiversity measure used here that does not incorporate notions of complementarity, which likely explains why these areas do not turn up in this one result. While the Barren Islands have received conservation attention in recent years, to our knowledge the other areas have not, and this convergence in prioritization among methods warrants additional field investigations.

### Comparing results: threats, opportunities and benefits

Each approach comes with a distinct set of strengths and weaknesses. Although not strictly a planning approach per se, the RGB maps are valuable for providing a rapid visual overview of the distribution of input variables and their interactions. The primary intent of this technique was descriptive, not prescriptive. Therefore, translating these simple RGB maps into specific management actions is challenging. The Marxan result, weighted Zonation result, and the categorical management action classification, on the other hand, can be used to recommend specific management and conservation actions, but based on different assumptions. As configured here, the Marxan solution shows areas that meet species representation targets set by analysts, maximize persistence in the face of environmental exposure, and reduce impacts on fisheries by minimizing the opportunity costs from foregone fishing revenues. The weighted Zonation result also shows areas that maximize species representation while avoiding areas of high exposure and fishing pressure. Unlike Marxan, however, Zonation produces a continuous ranking of biodiversity. In Zonation, therefore, it is up to users to decide which rank, or solution area, to choose as a threshold for a final result. In Marxan, by contrast, it is up to users to set targets for representation. Typically, Marxan and Zonation results such as these are used to guide decisions concerning the placement of strictly protected reserves [25], [74]. Because both the Marxan and the weighted Zonation results provide little information on the value, management and use of areas outside of those selected as top conservation priorities, however, they have less utility as comprehensive zoning tools.

The value of the categorical management action classification is that it can guide the placement of strict marine reserves and also zone the entire planning region into a variety of management categories that include various actions aimed at achieving more sustainable use of the seascape [70]. Zoning the entire area allows flexibility to consider management of “suboptimal” areas that would not be selected under optimization approaches, including degraded areas where effective management may lead to recovery. For example, maintaining herbivores through reduced fishing may help reefs recover from damage due to climate disturbances or destructive fishing [75]. The disadvantage of this method relative to optimization methods, however, is that they are likely to pick regions for strict conservation less efficiently, by not meeting biodiversity representation targets at minimum cost [76].

Fishing is a factor that has often undermined the success of protected area management [77], [78]. Despite this, it is frequently treated only as a factor to avoid in marine conservation planning. For example, Marxan is routinely configured to minimize fisheries conflicts and opportunity costs by avoiding areas of high fishing value [23]. However, there can be equally valid reasons for prioritizing protection of heavily fished areas because they may be areas of high productivity and source populations [79] that could stimulate spillover to fisheries [80], [81], and because closures can have their greatest fisheries benefits when fishing is beyond a maximum sustained yield [7], [82], [83]. Reserve networks that exclude fishing may not provide the economic benefits necessary for social adoption nor improve management more than standard fisheries management tools [84].

Many other ecosystem goods and services can be threatened by unsustainable management, and improved spatial planning tools are needed to handle this complexity. Emerging software, such as Marxan with Zones [67] and Marine InVEST [85], are promising. We did not compare either of these programs to the categorical management action classification because of our primary interest in evaluating Marxan and Zonation, the most widely used conservation planning tools in the region. Future efforts could consider a broader array of available and emerging approaches, however.

### Comparing results: impacts on fisheries and persistence

Marine spatial plans are frequently evaluated for their efficiency in meeting conservation objectives while minimizing economic impacts [72]. Given the assumption of no spillover and that strict protection completely displaces fishing activities, the impact of the large closures result on fisheries was ∼55% less than the Marxan 30% result. This difference in economic impact is explained by differences in the area of the two results. The large closures result is half the size of the Marxan area and represents significantly less of the total distribution of each species, on average. Importantly, there is little difference in economic impact between methods when Marxan targets are reduced to match the species proportions covered by the large closures result (Table 4). The Zonation 16% result, on the other hand, is about 3–4% more costly in terms of fisheries impacts than the other two results that also achieve about 16% representation. Giving additional negative weight to this factor in Zonation could potentially reduce this difference.

Spatial overlap between the three 16% representation results is relatively low (Figure 9). Despite similarities in cost and species proportions represented, the three results only share 9% of their total selected area. Two factors may explain these differences. One, according to our models, species beta-diversity is relatively low across the study area. When beta-diversity is low, many areas have similar species compositions, and as a consequence, many different potential reserve configurations can achieve roughly equal representation. Two, the method Marxan uses to incorporate the exposure variable is quite different from the other approaches. Marxan minimizes exposure probabilities across conservation features (species, habitats, bioregions), in balance with other costs and penalties, whereas the large closure and weighted Zonation results tend to avoid areas of high exposure entirely. Comparing raw exposure values, the Zonation 16% result consequently has the lowest mean exposure, followed by the large closure result, then the two Marxan results (Table 4). The exposure distributions from both the Zonation 16% result and the large closure result are significantly different from each other and the Marxan results. On the surface, the fact that exposure probability is lower in the large closures and Zonation 16% results than the Marxan optimizations is surprising. Marxan, however, seeks solutions that minimize exposure probability in order to meet representation targets for each individual conservation feature [28]. As a result, the probability of the large closures result missing feature targets is higher than both Marxan results, and highest in the Zonation 16% result (Table 4).

### Targets

By reducing Marxan representation targets to 16.3% to match the target level implied by the large closures result, we show that a classification-based method performs comparably to Marxan in terms of species representation and efficiency, though not as well in terms of the persistence of conservation features (Table 4). Similarly, we show that a 16% representation threshold on the weighted Zonation result produces comparable results to the other methods. Although necessary to compare approaches, this neglects an important question: what are adequate representation targets for marine spatial planning in Madagascar? While iterative, target-based approaches ensure species representation at the specified level [86], little is known about what constitutes an adequate target area for marine organisms [87]. For example, The Convention on Biological Diversity recommends “at least 10% of each of the world's marine and coastal ecological regions be effectively conserved by 2010” [88], while other organizations call for two to three times this level [89]. Targets used here fall within the broad range of these recommendations, but neither are adequately informed by species requirements. For example, many large migratory species such as sea turtles and cetaceans have specific breeding and nesting requirements. Protecting a portion of their local distribution may fail to protect these species over the long term unless augmented by ecologically meaningful guidelines across their distributional range [90]. Consequently, future national level marine conservation planning in Madagascar will benefit from a more rigorous elaboration of conservation targets, based on a comprehensive review and understanding of individual species life history traits, area requirements, and sources of mortality.

### Improvements and next steps

We expect marine spatial planning in Madagascar to improve with the availability of additional biological and environmental data. For example, datasets currently unavailable at sufficient resolution across the planning region include data on seagrass communities, information on habitat condition and intactness, site connectivity, and potential climate-induced acidification. Further, predicted responses, willingness to adopt management recommendations, and adaptive capacity of people to climate change is needed [70].

The Government of Madagascar committed to a plan to increase MPA coverage by at least one million hectares in 2003 [91], and this work is expected to continue into 2012 despite political changes resulting from political unrest in 2009. Consequently, the work presented here provides a foundation for the national level analyses needed to fulfill that target. There is considerable overlap in the areas identified for the highest priority reserves and, as the national discussion and management evolves, these methods and outputs can further help guide these decisions. Because implementation is both a political and a scientific process, however [92], [93], ultimately, the choice of approach should consider both goals for representation and persistence as well as the likelihood of adoption and compliance [78], [94], [95], [96], and the timing of implementation [97]. With this study, we show that methods based on Zonation and a simple combination of variables can produce result for strict protection that are similarly representative and have similar economic impacts as ones based on optimization. More broadly, we demonstrate the utility of comparing alternative methods early in the planning process for understanding patterns and interactions of key biodiversity and conservation variables.

## Supporting Information

Materials S1 **This text provides additional information on methods including modeling fish species distributions with MaxEnt, details of Marxan and Zonation runs, measuring representation of conservation features across results, and measuring exposure.**
(DOC)

Table S1 **Focal fish species list.** This list shows the 274 fish species with distributions modeled in MaxEnt for use in the analysis and data sources. OBIS refers to the Ocean Biogeographic Information System (http://www.iobis.org/). WCS, WWF, and CI refer to data provided by Wildlife Conservation Society, World Wildlife Fund, and Conservation International, respectively. COOKE refers to data provided by Andrew Cooke, and SAMOILY refers to data provided by Dr. Melita Samoily. Species marked with an asterix (*) were not included in the Marxan analyses because their continuous distribution value was below threshold.
(DOC)
