# Supplementary material for: Comparison of Marine Spatial Planning Methods in Madagascar Demonstrates Value of Alternative Approaches
Source: PLoS One. 2012 Feb 16;7(2):e28969. doi: 10.1371/journal.pone.0028969 (PMC3281012; doi:10.1371/journal.pone.0028969)
Supplement: Materials S1 — This text provides additional information on methods including modeling fish species distributions with MaxEnt, details of Marxan and Zonation runs, measuring representation of conservation features across results, and measuring exposure. (DOC) [file pone.0028969.s001.doc]

**Supplementary materials text (S1) for: Comparison of Marine Spatial Planning Methods in Madagascar Demonstrates Value of Alternative Approaches**

# Supplementary Methods

**Modeling fish species distributions with MaxEnt**

We used MaxEnt (version 3.3.2) [1] to create species distribution models for all species with at least 8 unique distribution records. Table S1 lists the 274 modeled species and sources. There were 8,702 occurrence (presence) records in total. We chose a 20 percent random test percentage, removed duplicate presence records, and selected 10,000 background points for model training. For remaining parameters and options, we maintained default values. For the resulting models, average training area under the curve (AUC) score was 0.9607, min AUC was 0.7683, and max AUC was 0.9998.

**Details of Zonation runs**

As noted in the methods, we ran Zonation twice, and used the results in two ways. First, we ran Zonation (version 2.1) [2] to map biodiversity value. The only inputs were biodiversity distributions, and included the following species and habitats in ESRI ASCII grid format: 274 continuous fish species distribution models (Table S1), 16 reef geomorphological types [3], mangroves [4,5], and three bioregions [6] (Table 2). We ran original core area Zonation, without any weights, boundary quality penalty, or smoothing. We did not choose the option to remove only from edges (cells could be removed from anywhere). We used a warp factor of one. For remaining parameters and options, we maintained default values.

Second, we ran Zonation (version 3.0.2) [7] to map areas of biodiversity value in balance with competing land uses. Input data included the following positively weighted features: 296 species and habitats in ESRI ASCII grid format: 274 fish species distribution models (Table S1), 16 reef geomorphological types [3], mangroves [4,5], and three bioregions [6], for a total of 294 features. In addition, we included the following negatively weighted features: fishing pressure (see methods) and exposure [8]. Each feature was weighted 1/294 (.003), thereby giving equal weight to all positively weighted features in balance with negatively weighted features, each of which was given a weight of one [7]. As in the first Zonation run, described above, we ran original core area Zonation, without boundary quality penalty, or smoothing. We did not choose the option to remove only from edges (cells could be removed from anywhere). We used a warp factor of one. For remaining parameters and options, we maintained default values.

**Marxan run details**

We used 32-bit Marxan (version 2.11) [9] to map areas to meet representation targets for conservation features. Conservation features included 251 fish species distribution models (Table S1), 16 reef geomorphological types [3], mangroves [4,5], and three bioregions [6], for a total of 271 features. Note that after thresholding continuous fish species distribution models (see methods), 23 species were removed because they no longer showed any distribution along in the study area. We ran Marxan twice, first with a single target of 30% for all species, habitats and bioregions, and second, with a feature target of 16.3% for all species, habitats and bioregions. All features were given a species penalty factor of 100. We set cost equal to modeled fish pressure per grid cell (described in methods), scaled to a range of 0-100. We set probability equal to exposure [8]. For both scenarios, we used the following parameters. Iterations: 1,000,000,000; runs: 100; boundary length modifier: 0.00005; probability weighting: 0.025.

**Measuring representation of conservation features across results**

We measured the average proportion of all conservation features (species, habitats, bioregions) represented by each of the three results: the Marxan 16% target scenario, the Marxan 30% target scenario, and the large closures categorical classification result. To measure average proportion represented, we used a custom script written in the ruby programming language to compare each result to the maps for habitats, bioregions and the full (not thresholded) set of 274 fish species distribution models (Table S1). The script compared each grid cell in each result to each habitat, bioregion and species distribution model, and summed the area (in the case of habitats or bioregions) or model values (in the case of fish species) across the results. This was compared to the total sum of area or model values across each map or model, to give a measure of the proportional “amount” of distribution included in each result. For example, a given result contains three grid cells with mangrove. Cell 1 has 400 m^2^, cell 2 has 300 m^2^ and cell 3 has 300 m^2^ of mangrove. Total mangrove distribution across the study area is 10,000 m^2^. In total, the solution represents 10% of the mangroves. For each result, this proportion was then averaged across habitats, bioregions and species as shown in Table 4.

**Measuring exposure**

We measured each result’s environmental exposure probability in two ways. First, we used the Wilcoxon rank-sum test to compare distribution of exposure values across each result, comparing the Marxan 30% result to the large closures result, and the Marxan 16% result to the large closures result. Second, we report the probability of each result not meeting feature targets, This is a statistic reported by the Marxan program for each run [10]. To compare the Marxan runs to the other results, we have to calculate this statistic for the large-closures and the Zonation 16% results. To do this, we select the set of planning units that exactly matches these results. We then “locked” these planning units into Marxan runs by setting “status” equal to two for these planning units. We left all other settings and exposure values identical to the regular Marxan runs. This effectively forces Marxan to select the exact same areas as those in the large closure and Zonation 16% result, and calculates the probability of these results not meeting feature targets, allowing us to compare this value between all results.

Supplementary References

1. Phillips SJ, Anderson RP, Schapire RE (2006) Maximum entropy modeling of species geographic distributions. Ecological Modelling 190: 231-259.

2. Moilanen A (2007) Landscape Zonation, benefit functions and target-based planning: Unifying reserve selection strategies. Biological Conservation 134.

3. Andréfouët S, Chagnaud N, Kranenburg CJ (2009) Atlas of Western Indian Ocean coral reefs. Centre IRD de Nouméa, New-Caledonia. 157 pages.

4. Harper GJ, Steininger MK, Tucker CJ, Juhn D, Hawkins F (2007) Fifty years of deforestation and forest fragmentation in Madagascar. Environmental Conservation 34: 325-333.

5. Moat J, Smith P (2007) Atlas of the vegetation of Madagascar. Kew: Kew Publishing, Royal Botanic Gardens.

6. Indian Ocean Commission (2010) A regional strategy for conserving marine ecosystems and fisheries of the Western Indian Ocean Islands Marine Ecoregion (WIOMER). Draft Report. Quatre Bornes, Mauritius and Antananarivo, Madagascar: Indian Ocean Commission and World Wildlife Fund.

7. Moilanen A, Anderson BJ, Eigenbrod F, Heinemeyer A, Roy DB, et al. (2011) Balancing alternative land uses in conservation prioritization. Ecological Applications 21: 1419-1426.

8. Maina J, Venus V, McClanahan TR, Ateweberhan M (2008) Modelling susceptibility of coral reefs to environmental stress using remote sensing data and GIS models. Ecological Modelling 212: 180-199.

9. Ball IR, Possingham HP, Watts ME (2009) Marxan and relatives: software for spatial conservation prioritization. In: Moilanen A, Possingham HP, Wilson KA, editors. Spatial conservation prioritization: Quantitative methods and computational tools. Oxford: Oxford University Press. pp. 185–195.

10. Watts M, Kircher L (2009) Advanced Marxan course manual. Brisbane, Queensland: The Ecology Centre, University of Queensland. 39 p.
