# Supplementary material for: Comparison of Marine Spatial Planning Methods in Madagascar Demonstrates Value of Alternative Approaches
Source: PLoS One. 2012 Feb 16;7(2):e28969. doi: 10.1371/journal.pone.0028969 (PMC3281012; doi:10.1371/journal.pone.0028969)
Supplement: Table S1 — Focal fish species list. This list shows the 274 fish species with distributions modeled in MaxEnt for use in the analysis and data sources. OBIS refers to the Ocean Biogeographic Information System (http://www.iobis.org/). WCS, WWF, and CI refer to data provided by Wildlife Conservation Society, World Wildlife Fund, and Conservation International, respectively. COOKE refers to data provided by Andrew Cooke, and SAMOILY refers to data provided by Dr. Melita Samoily. Species marked with an asterix (*) were not included in the Marxan analyses because their continuous distribution value was below threshold. (DOC) [file pone.0028969.s002.doc]

Table S1.

| **Species** | **Class** | **Order** | **Family** | **Data sources** |
| --- | --- | --- | --- | --- |
| Abudefduf vaigiensis | Actinopterygii | Perciformes | Pomacentridae | OBIS 2010, WCS 2008 |
| Acanthocybium solandri | Actinopterygii | Perciformes | Scombridae | OBIS 2010, WCS 2008 |
| Acanthurus leucosternon | Actinopterygii | Perciformes | Acanthuridae | OBIS 2010, WCS 2008 |
| Acanthurus lineatus | Actinopterygii | Perciformes | Acanthuridae | OBIS 2010, WCS 2008 |
| Aethaloperca rogaa | Actinopterygii | Perciformes | Serranidae | OBIS 2010, WCS 2008 |
| Aetobatus narinari | Chondrichthyes | Rajiformes | Myliobatidae | OBIS 2010, WCS 2008, COOKE 1995 |
| Alopias superciliosus | Chondrichthyes | Lamniformes | Alopiidae | OBIS 2010, COOKE 1995 |
| Amanses scopas | Actinopterygii | Tetraodontiformes | Monacanthidae | OBIS 2010, WCS 2008 |
| Ambassis natalensis | Actinopterygii | Perciformes | Ambassidae | OBIS 2010 |
| Amphiprion latifasciatus | Actinopterygii | Perciformes | Pomacentridae | WCS 2008, OBIS 2010 |
| Anacanthobatis marmoratus | Chondrichthyes | Rajiformes | Anacanthobatidae | OBIS 2010 |
| Anampses twistii | Actinopterygii | Perciformes | Labridae | OBIS 2010, WCS 2008 |
| Anguilla marmorata | Actinopterygii | Anguilliformes | Anguillidae | OBIS 2010, WCS 2008 |
| Anguilla mossambica | Actinopterygii | Anguilliformes | Anguillidae | OBIS 2010 |
| Antennarius coccineus | Actinopterygii | Lophiiformes | Antennariidae | OBIS 2010, WCS 2008 |
| Anyperodon leucogrammicus | Actinopterygii | Perciformes | Serranidae | OBIS 2010, WCS 2008 |
| Aphareus furca | Actinopterygii | Perciformes | Lutjanidae | OBIS 2010, WCS 2008 |
| Apolemichthys trimaculatus | Actinopterygii | Perciformes | Pomacanthidae | OBIS 2010, WCS 2008 |
| Aprion virescens | Actinopterygii | Perciformes | Lutjanidae | OBIS 2010, WCS 2008, CI 2007 |
| Argyrops filamentosus | Actinopterygii | Perciformes | Sparidae | OBIS 2010, WCS 2008 |
| Arothron hispidus | Actinopterygii | Perciformes | Tetraodontidae | OBIS 2010, WCS 2008 |
| Arothron mappa | Actinopterygii | Perciformes | Tetraodontidae | OBIS 2010, WCS 2008 |
| Arothron meleagris | Actinopterygii | Perciformes | Tetraodontidae | OBIS 2010, WCS 2008 |
| Arothron nigropunctatus | Actinopterygii | Perciformes | Tetraodontidae | OBIS 2010, WCS 2008 |
| Arothron stellatus | Actinopterygii | Perciformes | Tetraodontidae | OBIS 2010, WCS 2008 |
| Atule mate | Actinopterygii | Perciformes | Carangidae | OBIS 2010, WCS 2008 |
| Balistapus undulatus | Actinopterygii | Tetraodontiformes | Balistidae | OBIS 2010, WCS 2008 |
| Bodianus anthioides | Actinopterygii | Perciformes | Labridae | OBIS 2010, WCS 2008 |
| Bodianus diana | Actinopterygii | Perciformes | Labridae | OBIS 2010, WCS 2008 |
| Bothus mancus | Actinopterygii | Pleuronectiformes | Bothidae | OBIS 2010, WCS 2008 |
| Bothus pantherinus | Actinopterygii | Pleuronectiformes | Bothidae | OBIS 2010, WCS 2008 |
| Butis butis | Actinopterygii | Perciformes | Eleotridae | OBIS 2010 |
| Caesio caerulaurea | Actinopterygii | Perciformes | Caesionidae | OBIS 2010, WCS 2008 |
| Calotomus carolinus | Actinopterygii | Perciformes | Scaridae | OBIS 2010, WCS 2008 |
| Canthigaster janthinoptera | Actinopterygii | Perciformes | Tetraodontidae | OBIS 2010, WCS 2008 |
| Caracanthus madagascariensis | Actinopterygii | Scorpaeniformes | Caracanthidae | OBIS 2010, WCS 2008 |
| *Carangoides armatus | Actinopterygii | Perciformes | Carangidae | OBIS 2010, WCS 2008 |
| Carangoides chrysophrys | Actinopterygii | Perciformes | Carangidae | OBIS 2010, WCS 2008 |
| Carangoides gymnostethus | Actinopterygii | Perciformes | Carangidae | OBIS 2010 |
| Caranx ignobilis | Actinopterygii | Perciformes | Carangidae | OBIS 2010, WCS 2008, CI 2007 |
| Caranx melampygus | Actinopterygii | Perciformes | Carangidae | OBIS 2010, WCS 2008 |
| Caranx sexfasciatus | Actinopterygii | Perciformes | Carangidae | OBIS 2010, WCS 2008 |
| Carcharhinus albimarginatus | Chondrichthyes | Carcharhiniformes | Carcharhinidae | OBIS 2010 |
| Centrophorus moluccensis | Chondrichthyes | Squaliformes | Centrophoridae | OBIS 2010 |
| Cephalopholis argus | Actinopterygii | Perciformes | Serranidae | OBIS 2010, WCS 2008, CI 2007 |
| Cephalopholis aurantia | Actinopterygii | Perciformes | Serranidae | OBIS 2010, WCS 2008 |
| Cephalopholis boenak | Actinopterygii | Perciformes | Serranidae | OBIS 2010, WCS 2008, CI 2007 |
| Cephalopholis leopardus | Actinopterygii | Perciformes | Serranidae | OBIS 2010, WCS 2008 |
| Cephalopholis miniata | Actinopterygii | Perciformes | Serranidae | OBIS 2010, WCS 2008 |
| Cephalopholis sexmaculata | Actinopterygii | Perciformes | Serranidae | OBIS 2010, WCS 2008 |
| Cephalopholis sonnerati | Actinopterygii | Perciformes | Serranidae | OBIS 2010, WCS 2008 |
| Cephalopholis spiloparaea | Actinopterygii | Perciformes | Serranidae | OBIS 2010, WCS 2008 |
| Cephalopholis urodeta | Actinopterygii | Perciformes | Serranidae | OBIS 2010, WCS 2008 |
| Chaetodon auriga | Actinopterygii | Perciformes | Chaetodontidae | OBIS 2010, WCS 2008 |
| *Chaetodon blackburnii | Actinopterygii | Perciformes | Chaetodontidae | OBIS 2010 |
| Chaetodon guttatissimus | Actinopterygii | Perciformes | Chaetodontidae | OBIS 2010, WCS 2008 |
| Chaetodon interruptus | Actinopterygii | Perciformes | Chaetodontidae | OBIS 2010, WCS 2008 |
| Chaetodon kleinii | Actinopterygii | Perciformes | Chaetodontidae | OBIS 2010, WCS 2008 |
| Chaetodon madagaskariensis | Actinopterygii | Perciformes | Chaetodontidae | OBIS 2010, WCS 2008 |
| Chaetodon melannotus | Actinopterygii | Perciformes | Chaetodontidae | OBIS 2010, WCS 2008 |
| Chaetodon meyeri | Actinopterygii | Perciformes | Chaetodontidae | OBIS 2010, WCS 2008, WWF 2005 |
| Chaetodon trifascialis | Actinopterygii | Perciformes | Chaetodontidae | OBIS 2010, WCS 2008 |
| Chaetodon trifasciatus | Actinopterygii | Perciformes | Chaetodontidae | OBIS 2010, WCS 2008 |
| Chaetodon vagabundus | Actinopterygii | Perciformes | Chaetodontidae | OBIS 2010, WCS 2008 |
| Chaetodon xanthocephalus | Actinopterygii | Perciformes | Chaetodontidae | OBIS 2010, WCS 2008 |
| Chaetodon zanzibarensis | Actinopterygii | Perciformes | Chaetodontidae | OBIS 2010, WCS 2008 |
| *Chanos chanos | Actinopterygii | Gonorhynchiformes | Chanidae | OBIS 2010, WCS 2008 |
| Cheilinus chlorourus | Actinopterygii | Perciformes | Labridae | OBIS 2010, WCS 2008 |
| Cheilinus oxycephalus | Actinopterygii | Perciformes | Labridae | OBIS 2010, WCS 2008 |
| Cheilinus trilobatus | Actinopterygii | Perciformes | Labridae | OBIS 2010, WCS 2008, CI 2007 |
| Cheilinus undulatus | Actinopterygii | Perciformes | Labridae | OBIS 2010, WCS 2008, WCS 2008, CI 2007 |
| Cheilodipterus lineatus | Actinopterygii | Perciformes | Apogonidae | OBIS 2010, WCS 2008 |
| Chromis atripectoralis | Actinopterygii | Perciformes | Pomacentridae | WCS 2008, OBIS 2010 |
| Chromis dimidiata | Actinopterygii | Perciformes | Pomacentridae | OBIS 2010, WCS 2008, WWF 2005 |
| Chromis nigrura | Actinopterygii | Perciformes | Pomacentridae | OBIS 2010, WCS 2008 |
| Chromis ternatensis | Actinopterygii | Perciformes | Pomacentridae | OBIS 2010, WCS 2008 |
| Chromis viridis | Actinopterygii | Perciformes | Pomacentridae | OBIS 2010, WCS 2008 |
| Cirrhitus pinnulatus | Actinopterygii | Perciformes | Cirrhitidae | OBIS 2010, WCS 2008 |
| *Coryogalops anomolus | Actinopterygii | Perciformes | Gobiidae | OBIS 2010, WCS 2008 |
| *Coryphaena hippurus | Actinopterygii | Perciformes | Coryphaenidae | OBIS 2010, WCS 2008 |
| Dalatias licha | Chondrichthyes | Squaliformes | Dalatiidae | OBIS 2010 |
| Dascyllus aruanus | Actinopterygii | Perciformes | Pomacentridae | OBIS 2010, WCS 2008 |
| Dascyllus carneus | Actinopterygii | Perciformes | Pomacentridae | OBIS 2010, WCS 2008 |
| *Dasyatis kuhlii | Chondrichthyes | Rajiformes | Dasyatidae | OBIS 2010, WCS 2008, COOKE 1995 |
| Dinematichthys iluocoeteoides | Actinopterygii | Ophidiiformes | Bythitidae | OBIS 2010 |
| Dipturus crosnieri | Chondrichthyes | Rajiformes | Rajidae | OBIS 2010 |
| *Drepane punctata | Actinopterygii | Perciformes | Drepanidae | OBIS 2010, WCS 2008 |
| Echeneis naucrates | Actinopterygii | Perciformes | Echeneidae | OBIS 2010, WCS 2008 |
| Ecsenius midas | Actinopterygii | Perciformes | Blenniidae | OBIS 2010, WCS 2008 |
| Elops machnata | Actinopterygii | Elopiformes | Elopidae | OBIS 2010 |
| Encheliophis gracilis | Actinopterygii | Ophidiiformes | Carapidae | OBIS 2010 |
| Enneapterygius tutuilae | Actinopterygii | Perciformes | Tripterygiidae | OBIS 2010 |
| *Entomacrodus vermiculatus | Actinopterygii | Perciformes | Blenniidae | OBIS 2010, WCS 2008 |
| Epibulus insidiator | Actinopterygii | Perciformes | Labridae | OBIS 2010, WCS 2008 |
| *Epinephelus albomarginatus | Actinopterygii | Perciformes | Serranidae | OBIS 2010 |
| *Epinephelus andersoni | Actinopterygii | Perciformes | Serranidae | OBIS 2010 |
| Epinephelus areolatus | Actinopterygii | Perciformes | Serranidae | OBIS 2010, WCS 2008 |
| Epinephelus chlorostigma | Actinopterygii | Perciformes | Serranidae | OBIS 2010, WCS 2008 |
| Epinephelus fasciatus | Actinopterygii | Perciformes | Serranidae | OBIS 2010, WCS 2008 |
| Epinephelus flavocaeruleus | Actinopterygii | Perciformes | Serranidae | OBIS 2010, WCS 2008 |
| Epinephelus fuscoguttatus | Actinopterygii | Perciformes | Serranidae | OBIS 2010, WCS 2008 |
| Epinephelus hexagonatus | Actinopterygii | Perciformes | Serranidae | OBIS 2010, WCS 2008 |
| Epinephelus lanceolatus | Actinopterygii | Perciformes | Serranidae | OBIS 2010, WCS 2008 |
| Epinephelus longispinis | Actinopterygii | Perciformes | Serranidae | OBIS 2010, WCS 2008 |
| Epinephelus macrospilos | Actinopterygii | Perciformes | Serranidae | OBIS 2010, WCS 2008 |
| Epinephelus malabaricus | Actinopterygii | Perciformes | Serranidae | OBIS 2010, WCS 2008 |
| *Epinephelus marginatus | Actinopterygii | Perciformes | Serranidae | OBIS 2010 |
| Epinephelus melanostigma | Actinopterygii | Perciformes | Serranidae | OBIS 2010, WCS 2008 |
| Epinephelus merra | Actinopterygii | Perciformes | Serranidae | OBIS 2010, WCS 2008, CI 2007 |
| Epinephelus morrhua | Actinopterygii | Perciformes | Serranidae | OBIS 2010, WCS 2008 |
| Epinephelus multinotatus | Actinopterygii | Perciformes | Serranidae | OBIS 2010, WCS 2008 |
| Epinephelus ongus | Actinopterygii | Perciformes | Serranidae | OBIS 2010, WCS 2008 |
| Epinephelus poecilonotus | Actinopterygii | Perciformes | Serranidae | OBIS 2010 |
| Epinephelus rivulatus | Actinopterygii | Perciformes | Serranidae | OBIS 2010, WCS 2008 |
| Epinephelus spilotoceps | Actinopterygii | Perciformes | Serranidae | OBIS 2010, WCS 2008 |
| Epinephelus tauvina | Actinopterygii | Perciformes | Serranidae | OBIS 2010, WCS 2008 |
| Epinephelus tukula | Actinopterygii | Perciformes | Serranidae | OBIS 2010, WCS 2008 |
| Eridacnis sinuans | Chondrichthyes | Carcharhiniformes | Proscyllidae | OBIS 2010 |
| Etmopterus sentosus | Chondrichthyes | Squaliformes | Etmopteridae | OBIS 2010 |
| Eurypegasus draconis | Actinopterygii | Gasterosteiformes | Pegasidae | OBIS 2010 |
| *Euthynnus affinis | Actinopterygii | Perciformes | Scombridae | OBIS 2010, WCS 2008 |
| Eviota distigma | Actinopterygii | Perciformes | Gobiidae | WCS 2008, OBIS 2010 |
| Eviota sebreei | Actinopterygii | Perciformes | Gobiidae | OBIS 2010 |
| Exallias brevis | Actinopterygii | Perciformes | Blenniidae | OBIS 2010, WCS 2008 |
| Forcipiger flavissimus | Actinopterygii | Perciformes | Chaetodontidae | OBIS 2010, WCS 2008 |
| Forcipiger longirostris | Actinopterygii | Perciformes | Chaetodontidae | OBIS 2010, WCS 2008 |
| Galeocerdo cuvier | Chondrichthyes | Carcharhiniformes | Carcharhinidae | OBIS 2010 |
| Gobiodon citrinus | Actinopterygii | Perciformes | Gobiidae | OBIS 2010, WCS 2008 |
| Gobiodon rivulatus | Actinopterygii | Perciformes | Gobiidae | OBIS 2010, WCS 2008 |
| Gomphosus caeruleus | Actinopterygii | Perciformes | Labridae | OBIS 2010, WCS 2008 |
| Gracila albomarginata | Actinopterygii | Perciformes | Serranidae | OBIS 2010, WCS 2008 |
| Gymnocranius grandoculis | Actinopterygii | Perciformes | Lethrinidae | OBIS 2010, WCS 2008 |
| Gymnosarda unicolor | Actinopterygii | Perciformes | Scombridae | OBIS 2010, WCS 2008 |
| Halaelurus lutarius | Chondrichthyes | Carcharhiniformes | Scyliorhinidae | OBIS 2010 |
| Halichoeres hortulanus | Actinopterygii | Perciformes | Labridae | OBIS 2010, WCS 2008 |
| Halichoeres iridis | Actinopterygii | Perciformes | Labridae | OBIS 2010, WCS 2008 |
| Helcogramma fuscopinna | Actinopterygii | Perciformes | Tripterygiidae | OBIS 2010, WCS 2008 |
| Hemigymnus fasciatus | Actinopterygii | Perciformes | Labridae | OBIS 2010, WCS 2008 |
| Hemiramphus far | Actinopterygii | Beloniformes | Hemiramphidae | OBIS 2010, WCS 2008 |
| Hemitaurichthys zoster | Actinopterygii | Perciformes | Chaetodontidae | OBIS 2010, WCS 2008 |
| Heniochus acuminatus | Actinopterygii | Perciformes | Chaetodontidae | OBIS 2010, WCS 2008 |
| Heniochus monoceros | Actinopterygii | Perciformes | Chaetodontidae | OBIS 2010, WCS 2008, WWF 2005 |
| Heptranchias perlo | Chondrichthyes | Hexanchiformes | Hexanchidae | OBIS 2010 |
| Herklotsichthys quadrimaculatus | Actinopterygii | Clupeiformes | Clupeidae | OBIS 2010, WCS 2008 |
| Heterodontus ramalheira | Chondrichthyes | Heterodontiformes | Heterodontidae | OBIS 2010 |
| Heteropriacanthus cruentatus | Actinopterygii | Perciformes | Priacanthidae | OBIS 2010, WCS 2008 |
| Hexanchus griseus | Chondrichthyes | Hexanchiformes | Hexanchidae | OBIS 2010, COOKE 1995 |
| *Hippocampus kuda | Actinopterygii | Syngnathiformes | Syngnathidae | OBIS 2010, WCS 2008 |
| Hipposcarus harid | Actinopterygii | Perciformes | Scaridae | OBIS 2010, WCS 2008 |
| Holohalaelurus punctatus | Chondrichthyes | Carcharhiniformes | Scyliorhinidae | OBIS 2010 |
| Istiophorus platypterus | Actinopterygii | Perciformes | Istiophoridae | OBIS 2010, WCS 2008 |
| Katsuwonus pelamis | Actinopterygii | Perciformes | Scombridae | OBIS 2010, WCS 2008 |
| Kuhlia mugil | Actinopterygii | Perciformes | Kuhliidae | OBIS 2010 |
| Kuhlia rupestris | Actinopterygii | Perciformes | Kuhliidae | OBIS 2010 |
| Labrichthys unilineatus | Actinopterygii | Perciformes | Labridae | OBIS 2010, WCS 2008 |
| Labropsis xanthonota | Actinopterygii | Perciformes | Labridae | WCS 2008, OBIS 2010 |
| Latimeria chalumnae | Sarcopterygii | Coelacanthiformes | Latimeriidae | OBIS 2010 |
| Lethrinus borbonicus | Actinopterygii | Perciformes | Lethrinidae | OBIS 2010, WCS 2008 |
| Lethrinus crocineus | Actinopterygii | Perciformes | Lethrinidae | OBIS 2010, WCS 2008 |
| Lethrinus harak | Actinopterygii | Perciformes | Lethrinidae | OBIS 2010, WCS 2008, CI 2007, SAMOILYS 2010 |
| Lethrinus mahsena | Actinopterygii | Perciformes | Lethrinidae | OBIS 2010, WCS 2008, CI 2007, SAMOILYS 2010 |
| Lethrinus microdon | Actinopterygii | Perciformes | Lethrinidae | WCS 2008, OBIS 2010 |
| Lethrinus nebulosus | Actinopterygii | Perciformes | Lethrinidae | OBIS 2010, WCS 2008, CI 2007 |
| Lethrinus rubrioperculatus | Actinopterygii | Perciformes | Lethrinidae | OBIS 2010, WCS 2008 |
| Lethrinus variegatus | Actinopterygii | Perciformes | Lethrinidae | OBIS 2010, WCS 2008 |
| Loxodon macrorhinus | Chondrichthyes | Carcharhiniformes | Carcharhinidae | OBIS 2010, COOKE 1995 |
| Lutjanus argentimaculatus | Actinopterygii | Perciformes | Ludjanidae | OBIS 2010, WCS 2008, CI 2007 |
| Lutjanus bohar | Actinopterygii | Perciformes | Lutjanidae | OBIS 2010, WCS 2008, CI 2007 |
| Lutjanus monostigma | Actinopterygii | Perciformes | Lutjanidae | OBIS 2010, WCS 2008, CI 2007 |
| Lutjanus notatus | Actinopterygii | Perciformes | Lutjanidae | OBIS 2010, WCS 2008 |
| Lutjanus russellii | Actinopterygii | Perciformes | Lutjanidae | OBIS 2010 |
| Lutjanus sebae | Actinopterygii | Perciformes | Lutjanidae | OBIS 2010 |
| Macropharyngodon cyanoguttatus | Actinopterygii | Perciformes | Labridae | OBIS 2010, WCS 2008 |
| Malacanthus brevirostris | Actinopterygii | Perciformes | Malacanthidae | OBIS 2010, WCS 2008 |
| Malacanthus latovittatus | Actinopterygii | Perciformes | Malacanthidae | OBIS 2010, WCS 2008 |
| Megalops cyprinoides | Actinopterygii | Elopiformes | Megalopidae | OBIS 2010 |
| Melichthys indicus | Actinopterygii | Tetraodontiformes | Balistidae | OBIS 2010, WCS 2008 |
| Melichthys niger | Actinopterygii | Tetraodontiformes | Balistidae | OBIS 2010, WCS 2008 |
| Monotaxis grandoculis | Actinopterygii | Perciformes | Lethrinidae | OBIS 2010, WCS 2008, CI 2007 |
| Myripristis adusta | Actinopterygii | Beryciformes | Holocentridae | OBIS 2010, WCS 2008 |
| Myripristis berndti | Actinopterygii | Beryciformes | Holocentridae | OBIS 2010, WCS 2008 |
| Myripristis hexagona | Actinopterygii | Beryciformes | Holocentridae | OBIS 2010, WCS 2008 |
| Myripristis kuntee | Actinopterygii | Beryciformes | Holocentridae | OBIS 2010, WCS 2008 |
| Myripristis murdjan | Actinopterygii | Beryciformes | Holocentridae | OBIS 2010, WCS 2008 |
| Myripristis seychellensis | Actinopterygii | Beryciformes | Holocentridae | OBIS 2010, WCS 2008 |
| Myripristis vittata | Actinopterygii | Beryciformes | Holocentridae | OBIS 2010, WCS 2008 |
| *Nebrius ferrugineus | Chondrichthyes | Orectolobiformes | Ginglymostomatidae | OBIS 2010, WCS 2008, COOKE 1995 |
| Neoglyphidodon melas | Actinopterygii | Perciformes | Pomacentridae | OBIS 2010, WCS 2008 |
| Neoniphon sammara | Actinopterygii | Beryciformes | Holocentridae | OBIS 2010, WCS 2008 |
| Novaculichthys taeniourus | Actinopterygii | Perciformes | Labridae | OBIS 2010 |
| Odontaspis ferox | Chondrichthyes | Lamniformes | Odontaspididae | COOKE 1995, OBIS 2010 |
| Ophiocara porocephala | Actinopterygii | Perciformes | Eleotridae | OBIS 2010 |
| Ostracion cubicus | Actinopterygii | Tetraodontiformes | Ostraciidae | OBIS 2010, WCS 2008 |
| Ostracion meleagris | Actinopterygii | Tetraodontiformes | Ostraciidae | OBIS 2010, WCS 2008 |
| Oxymonacanthus longirostris | Actinopterygii | Tetraodontiformes | Monacanthidae | OBIS 2010, WCS 2008 |
| Paracaesio xanthura | Actinopterygii | Perciformes | Lutjanidae | OBIS 2010 |
| Paracanthurus hepatus | Actinopterygii | Perciformes | Acanthuridae | OBIS 2010 |
| Paracirrhites arcatus | Actinopterygii | Perciformes | Cirrhitidae | OBIS 2010, WCS 2008 |
| Paracirrhites forsteri | Actinopterygii | Perciformes | Cirrhitidae | OBIS 2010, WCS 2008 |
| Paragobiodon echinocephalus | Actinopterygii | Perciformes | Gobiidae | OBIS 2010, WCS 2008 |
| Parupeneus indicus | Actinopterygii | Perciformes | Mullidae | OBIS 2010, WCS 2008 |
| Periophthalmus kalolo | Actinopterygii | Perciformes | Gobiidae | OBIS 2010 |
| Pervagor janthinosoma | Actinopterygii | Tetraodontiformes | Monacanthidae | OBIS 2010 |
| Plagiotremus rhinorhynchos | Actinopterygii | Perciformes | Blenniidae | OBIS 2010, WCS 2008 |
| Plagiotremus tapeinosoma | Actinopterygii | Perciformes | Blenniidae | OBIS 2010, WCS 2008 |
| Platax pinnatus | Actinopterygii | Perciformes | Ephippidae | OBIS 2010, WCS 2008 |
| Plectorhinchus gaterinus | Actinopterygii | Perciformes | Haemulidae | OBIS 2010, WCS 2008 |
| Plectorhinchus gibbosus | Actinopterygii | Perciformes | Haemulidae | OBIS 2010, WCS 2008 |
| Plectroglyphidodon dickii | Actinopterygii | Perciformes | Pomacentridae | OBIS 2010, WCS 2008 |
| Plectroglyphidodon imparipennis | Actinopterygii | Perciformes | Pomacentridae | OBIS 2010, WCS 2008 |
| Plectroglyphidodon johnstonianus | Actinopterygii | Perciformes | Pomacentridae | OBIS 2010, WCS 2008 |
| Plectropomus laevis | Actinopterygii | Perciformes | Serranidae | WCS 2008, OBIS 2010, CI 2007 |
| Plectropomus punctatus | Actinopterygii | Perciformes | Serranidae | OBIS 2010, WCS 2008, CI 2007, SAMOILYS 2010 |
| Plesiobatis daviesi | Mammalia | Rajiformes | Plesiobatidae | OBIS 2010 |
| Pliotrema warreni | Chondrichthyes | Pristiophoriformes | Pristiophoridae | OBIS 2010 |
| Pomacanthus imperator | Actinopterygii | Perciformes | Pomacanthidae | OBIS 2010, WCS 2008 |
| Pomacanthus semicirculatus | Actinopterygii | Perciformes | Pomacanthidae | OBIS 2010, WCS 2008 |
| Pomacentrus pikei | Actinopterygii | Perciformes | Pomacentridae | OBIS 2010, WCS 2008 |
| *Pomadasys furcatum | Actinopterygii | Perciformes | Haemulidae | OBIS 2010 |
| Priacanthus hamrur | Actinopterygii | Perciformes | Priacanthidae | OBIS 2010, WCS 2008, WWF 2005, CI 2007 |
| *Pseudobalistes fuscus | Actinopterygii | Tetraodontiformes | Balistidae | OBIS 2010, WCS 2008 |
| *Pseudocarcharias kamoharai | Chondrichthyes | Lamniformes | Pseudocarchariidae | OBIS 2010 |
| Pseudodax moluccanus | Actinopterygii | Perciformes | Labridae | OBIS 2010, WCS 2008 |
| *Rachycentron canadum | Actinopterygii | Perciformes | Rachycentridae | OBIS 2010 |
| Rastrelliger kanagurta | Actinopterygii | Perciformes | Scombridae | OBIS 2010 |
| Remora remora | Actinopterygii | Perciformes | Echeneidae | OBIS 2010, CI 2007 |
| Rhabdosargus sarba | Actinopterygii | Perciformes | Sparidae | OBIS 2010, WCS 2008 |
| Rhincodon typus | Chondrichthyes | Orectolobiformes | Rhincodontidae | OBIS 2010, WCS 2008 |
| Rhizoprionodon acutus | Chondrichthyes | Carcharhiniformes | Carcharhinidae | OBIS 2010 |
| Sargocentron caudimaculatum | Actinopterygii | Beryciformes | Holocentridae | OBIS 2010, WCS 2008, CI 2007 |
| Sargocentron diadema | Actinopterygii | Beryciformes | Holocentridae | OBIS 2010, WCS 2008 |
| Sargocentron inaequalis | Actinopterygii | Beryciformes | Holocentridae | OBIS 2010 |
| Sargocentron melanospilos | Actinopterygii | Beryciformes | Holocentridae | OBIS 2010, WCS 2008 |
| Sargocentron spiniferum | Actinopterygii | Beryciformes | Holocentridae | OBIS 2010, WCS 2008, CI 2007 |
| Sargocentron tiere | Actinopterygii | Beryciformes | Holocentridae | OBIS 2010 |
| Sargocentron tiereoides | Actinopterygii | Beryciformes | Holocentridae | OBIS 2010, WCS 2008 |
| Scarus caudofasciatus | Actinopterygii | Perciformes | Scaridae | WCS 2008, OBIS 2010 |
| Scarus falcipinnis | Actinopterygii | Perciformes | Scaridae | OBIS 2010, WCS 2008 |
| Scarus frenatus | Actinopterygii | Perciformes | Scaridae | OBIS 2010, WCS 2008 |
| Scarus ghobban | Actinopterygii | Perciformes | Scaridae | OBIS 2010, WCS 2008, CI 2007 |
| Scarus globiceps | Actinopterygii | Perciformes | Scaridae | OBIS 2010, WCS 2008 |
| Scarus psittacus | Actinopterygii | Perciformes | Scaridae | OBIS 2010, WCS 2008 |
| Scarus scaber | Actinopterygii | Perciformes | Scaridae | OBIS 2010, WCS 2008, CI 2007 |
| Scomberomorus commerson | Actinopterygii | Perciformes | Scombridae | OBIS 2010, CI 2007 |
| Scorpaenodes parvipinnis | Actinopterygii | Scorpaeniformes | Scorpaenidae | OBIS 2010, WCS 2008 |
| Selar crumenophthalmus | Actinopterygii | Perciformes | Carangidae | OBIS 2010, WCS 2008 |
| Sicyopterus lagocephalus | Actinopterygii | Perciformes | Gobiidae | OBIS 2010 |
| Sillago sihama | Actinopterygii | Perciformes | Sillaginidae | OBIS 2010, WCS 2008 |
| *Sphyraena acutipinnis | Actinopterygii | Perciformes | Sphyraenidae | OBIS 2010, WCS 2008 |
| Sphyraena barracuda | Actinopterygii | Perciformes | Sphyraenidae | OBIS 2010, WCS 2008 |
| Sphyraena obtusata | Actinopterygii | Perciformes | Sphyraenidae | OBIS 2010, WCS 2008 |
| *Sphyrna lewini | Chondrichthyes | Carcharhiniformes | Sphyrnidae | OBIS 2010, COOKE 1995 |
| Sphyrna mokarran | Chondrichthyes | Carcharhiniformes | Sphyrnidae | OBIS 2010, COOKE 1995 |
| Squalus megalops | Chondrichthyes | Squaliformes | Squalidae | OBIS 2010 |
| *Squalus mitsukurii | Chondrichthyes | Squaliformes | Squalidae | OBIS 2010 |
| Squatina africana | Chondrichthyes | Squatiniformes | Squatinidae | OBIS 2010 |
| Stegastes limbatus | Actinopterygii | Perciformes | Pomacentridae | OBIS 2010, WCS 2008 |
| Stegostoma fasciatum | Chondrichthyes | Orectolobiformes | Stegostomatidae | OBIS 2010, COOKE 1995, WCS 2008 |
| Strongylura leiura | Actinopterygii | Beloniformes | Belonidae | OBIS 2010, WCS 2008 |
| Syngnathoides biaculeatus | Actinopterygii | Syngnathiformes | Syngnathidae | OBIS 2010, WCS 2008 |
| *Taeniura lymma | Chondrichthyes | Rajiformes | Dasyatidae | OBIS 2010 |
| Terapon jarbua | Actinopterygii | Perciformes | Terapontidae | OBIS 2010, WCS 2008 |
| Thalassoma genivittatum | Actinopterygii | Perciformes | Labridae | OBIS 2010, WCS 2008 |
| Thalassoma hardwicke | Actinopterygii | Perciformes | Labridae | OBIS 2010, WCS 2008 |
| Thalassoma hebraicum | Actinopterygii | Perciformes | Labridae | OBIS 2010, WCS 2008 |
| Thryssa baelama | Actinopterygii | Clupeiformes | Engraulidae | OBIS 2010, WCS 2008 |
| *Thunnus albacares | Actinopterygii | Perciformes | Scombridae | OBIS 2010, WCS 2008 |
| Torpedo fuscomaculata | Chondrichthyes | Torpediniformes | Torpedinidae | OBIS 2010, COOKE 1995, WCS 2008 |
| Torpedo sinuspersici | Chondrichthyes | Torpediniformes | Torpedinidae | OBIS 2010, COOKE 1995, WCS 2008 |
| Trachinotus blochii | Actinopterygii | Perciformes | Carangidae | OBIS 2010, WCS 2008 |
| Tylosurus crocodilus | Actinopterygii | Beloniformes | Belonidae | WCS 2008, OBIS 2010 |
| Variola albimarginata | Actinopterygii | Perciformes | Serranidae | OBIS 2010, WCS 2008 |
| Variola louti | Actinopterygii | Perciformes | Serranidae | OBIS 2010, WCS 2008, CI 2007 |
| Zebrasoma scopas | Actinopterygii | Perciformes | Acanthuridae | OBIS 2010 |
